# Exact mass analysis of sulfur clusters upon encapsulation by a polyaromatic capsular matrix

**DOI:** 10.1038/s41467-017-00605-5

**Published:** 2017-09-29

**Authors:** Sho Matsuno, Masahiro Yamashina, Yoshihisa Sei, Munetaka Akita, Akiyoshi Kuzume, Kimihisa Yamamoto, Michito Yoshizawa

**Affiliations:** 1 Laboratory for Chemistry and Life Science, Institute of Innovative Research, Tokyo Institute of Technology, 4259 Nagatsuta, Midori-ku, Yokohama, 226-8503 Japan; 2 Present Address: Department of Chemistry, University of Cambridge, Lensfield Road, Cambridge, CB2 1EW UK

## Abstract

Structural determination of inorganic clusters relies heavily on mass spectrometry because of, in most cases, their poor responsivities toward nuclear magnetic resonance, ultraviolet/visible, and infrared analyses. Nevertheless, mass spectrometry analysis of oligosulfurs (S_*n*_), which are unique clusters with copious allotropic forms, usually displays their fragment peaks. Here we report that a polyaromatic capsule acts as a supramolecular matrix for the mass determination of the neutral sulfur clusters. Upon encapsulation, molecular ion peaks derived from the host–guest complexes including cyclic S_6_ and S_8_ clusters are exclusively detected by common electrospray ionization time-of-flight mass spectrometry analysis. Furthermore, mass spectrometry analysis of a cyclic S_12_ cluster, which is in situ prepared from two S_6_ clusters within the matrix upon light irradiation, is achieved by the same way. The present matrix can remarkably stabilize the otherwise labile S_6_ and S_12_ clusters in the polyaromatic shell not only under mass spectrometry conditions but also in an ambient solution state.

## Abstract (web summary)

The structures of inorganic clusters are commonly characterized by mass spectrometry (MS), but neutral sulfur clusters heavily fragment under MS conditions, preventing their exact mass determination. Here, the authors successfully perform MS on labile cyclic sulfur clusters by stabilizing them within ionic supramolecular capsules.

## Introduction

**Fig. 1:**
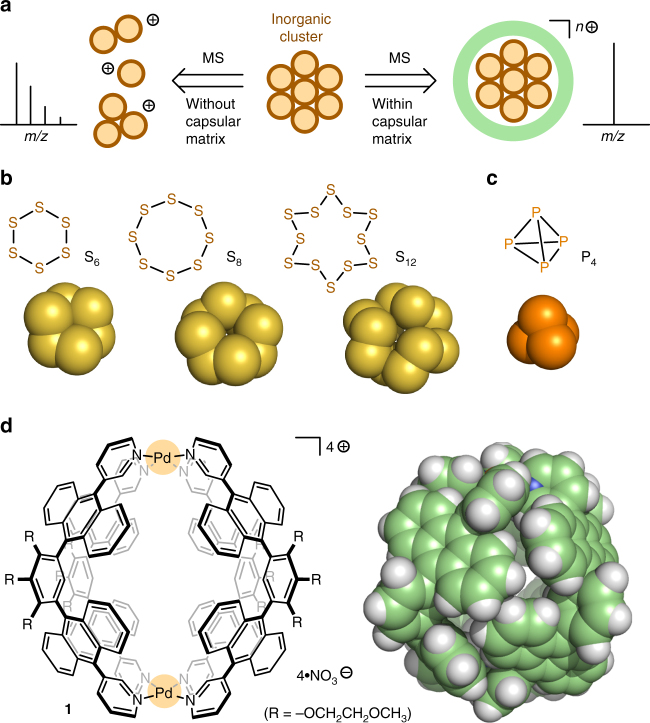
MS analysis of an inorganic cluster and structure of sulfur clusters and polyaromatic capsule **1**. **a** Schematic representation of the MS analysis of a neutral inorganic cluster within/without a capsular matrix and their typical ion peaks. **b** Sulfur clusters S_6_, S_8_, and S_12_ and their crystal or optimized structures. **c** White phosphorus and its crystal structure^37^. **d** Polyaromatic capsule **1** and the space-filling representation of the crystal structure (substituents and counterions are omitted for clarity)

Mass spectrometry (MS) is of particular importance for the structural characterization of inorganic clusters, because more precise information is not easily obtained by other analytical techniques such as nuclear magnetic resonance (NMR), ultraviolet-visible (UV/Vis), and infrared (IR) spectroscopies^1, 2^. However, most of the neutral clusters fully or partially decompose within MS^3^, which prevents us from determining the molecular weight of the target structures. Sulfur clusters continue to attract attention in the fields of physical and synthetic chemistry due to their unique structures and reactivities^4–7^. More than 30 allotropes of sulfur (e.g., S_6-15_) are known to date, yet mass determination of the uncharged structures has been virtually impossible by previous MS methods. Owing to the instability of oligosulfur ions generated under usual electron ionization conditions, immediate and substantial fragmentation reactions are typically observed (Fig. 1a, *left*). Therefore, no reliable method to obtain the structural information of such metastable clusters in solution has been reported. We anticipated that if the neutral sulfur clusters (Fig. 1b) were fully wrapped with an ionic capsular matrix, the exact molecular weights could be readily available on the MS analysis (Fig. 1a, *right*), because of the protection of the encapsulated clusters and the promotion of the efficient ionization. In addition, development of a new class of supramolecular matrixes for common mass spectrometry would assist in the creation of not only unknown sulfur clusters but also novel metalloclusters. There are many reports on the successful stabilization and observation of highly reactive organic and organometallic compounds within supramolecular cages and capsules^8–18^. Nevertheless, those of labile inorganic clusters are limited to white phosphorus (Fig. 1c)^19^ and yellow arsenic^20, 21^.

Here we report the MS analysis of cyclic sulfur clusters S_*n*_ (*n* = 6, 8, and 12; Fig. 1b) upon encapsulation. As an analytical matrix, we employ supramolecular capsule **1** (Fig. 1d)^22^ bearing a positively charged (4+) polyaromatic shell suitable for electrospray ionization time-of-flight (ESI-TOF) MS analysis^2^. The composition of the supramolecular matrix is simple, i.e., M_2_L_4_ with two Pd(II) ions and four bent bispyridine ligands with two anthracene panels^23^. The spherical cavity surrounded by the multiple polyaromatic panels can efficiently accommodate various organic molecules with dimensions of up to 1 nm (e.g., BODIPY, corannulene, and fullerene C_60_) in aqueous solutions through the hydrophobic effect and π-stacking interactions^18, 24–26^. However, solution-state host–guest interactions between the inorganic sulfur clusters and our capsules as well as other supramolecular cages remained uncertain so far^27, 28^. In the present work, we demonstrate the exact mass determination of cyclic S_6_ and S_8_ clusters by common ESI-TOF MS analysis upon encapsulation within capsular matrix **1**. In addition, we accomplish selective preparation of a cyclic S_12_ cluster from two S_6_ clusters within the matrix upon light irradiation, as revealed by subsequent MS analysis of the host–guest complex.

## Results

### MS analysis of a cyclic S_8_ cluster

**Fig. 2:**
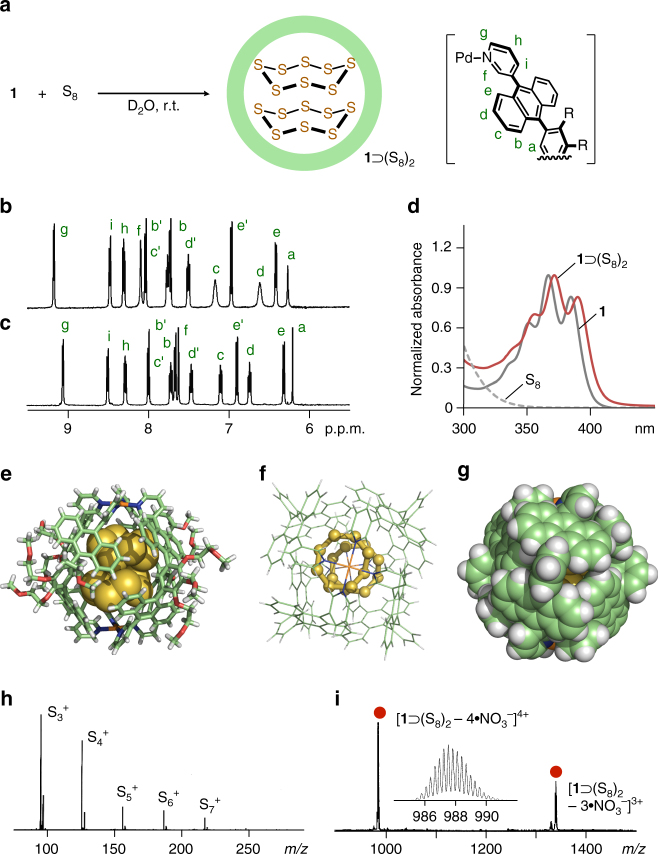
Formation and characterization of **1**⊃(S_8_)_2_. **a** Schematic representation of the encapsulation of two S_8_ clusters within matrix **1**. ^1^H NMR spectra (500 MHz, D_2_O, room temperature) of **b** **1** and **c** **1**⊃(S_8_)_2_. **d** UV-visible spectra (room temperature) of **1** and **1**⊃(S_8_)_2_ in H_2_O, and S_8_ in CH_3_OH. X-ray crystal structure of **1**⊃(S_8_)_2_ (host and guest parts): **e** cylinder and space-filling representation, **f** ball-and-stick, and **g** space-filling representations (the peripheral substituents are replaced by hydrogen atoms). **h** MALDI-TOF MS spectrum of S_8_. **i** ESI-TOF MS spectrum (H_2_O) of **1**⊃(S_8_)_2_ and the expansion of the [**1**⊃(S_8_)_2_−4*•*NO_3_^−^]^4+^ peak

We firstly carried out the quantitative encapsulation and mass determination of *cyclo*-octasulfur (S_8_) using capsular matrix **1** in water. Stirring excess hydrophobic S_8_ (3.9 μmol) in a D_2_O solution (0.5 ml) of **1** (0.40 μmol) at room temperature for 30 min led to the exclusive formation of a **1**⊃(S_8_)_2_ complex (Fig. 2a). After removal of suspended free S_8_ by filtration, the 1:2 host–guest structure was confirmed by NMR, UV-visible, and X-ray crystallographic analyses. In the ^1^H NMR spectrum, the appearance of new aromatic signals and the disappearance of the original matrix signals are indicative of the quantitative encapsulation of the sulfur cluster (Figs. 2b, c and see Supplementary Fig. 1). Due to the inclusion, the internal *H*_f_ signal of **1** was largely shifted upfield (Δ*δ* = −0.40 p.p.m.), whereas the external *H*_h_ signal remained almost unchanged. UV-visible spectrum of the product showed slight red-shifts (Δ*λ*_max_ = +6 nm) of the absorption bands derived from the anthracene moieties of **1** (Fig. 2d), indicating S-*π* interactions^29^. Direct evidence of the **1**⊃(S_8_)_2_ structure was obtained from the X-ray crystallographic analysis. Pale-yellow crystals suitable for X-ray analysis grew by the slow evaporation of a 25:1 H_2_O/CH_3_CN solution of the product at room temperature for 1 month. The molecular structure revealed that two molecules of S_8_ fully occupy the spherical cavity of **1** in a stacked fashion (Figs. 2e, f and see Supplementary Fig. 3). Each of the crown-shaped rings is in close proximity with the closest intermolecular S•••S distance of 3.1 Å. With regard to the host–guest interactions, the closest distances between the sulfur atoms and the eight anthracene panels are less than 3.6 Å (see Supplementary Fig. 4), whereas those between the sulfur atoms and the Pd(II) centers are more than 4.8 Å. The S_8_ dimer can be isolated from the bulk phase by the multiple anthracene panels of **1** (Fig. 2g).

Although the S_8_ cluster is the most stable sulfur allotrope^6^, the matrix-assisted laser desorption ionization (MALDI)-TOF MS spectrum displays several ion peaks derived from the fragments such as S_3_^+^, S_4_^+^, and S_5_^+^ species (*m/z* = 96, 128, and 160, respectively; Fig. 2h) even under various conditions^30^. ESI-TOF MS analysis of S_8_ also shows no target peak (see Supplementary Fig. 5). In contrast, ESI-TOF MS spectrum of the product showed prominent peaks at *m/z* = 988.0 and 1338.1, corresponding to 1:2 host–guest [**1**⊃(S_8_)_2_ – *n•*NO_3_^−^]^*n*+^ species (*n* = 4 and 3, respectively; Fig. 2i and see Supplementary Fig. 6). No MS peaks assignable to the empty matrix and host–guest complexes including decomposed S_8_ clusters were detected so that the significant stabilization of S_8_ under MS conditions was demonstrated upon encapsulation within **1**. The non-covalent host–guest structure remains intact in water even under highly diluted conditions (<5.0 μM; see Supplementary Fig. 7), suggesting the potential application for microgram-scale MS analysis. Interestingly, the binding is stronger than that of hydrophobic cyclooctane. ^1^H NMR competitive binding experiments revealed that the matrix encapsulates S_8_ clusters with >90% selectivity from a 1:1 mixture of the cyclic sulfur and alkane (20 equiv. each) under ambient aqueous conditions (see Supplementary Fig. 8).

### Stabilization and MS analysis of a cyclic S_6_ cluster

**Fig. 3:**
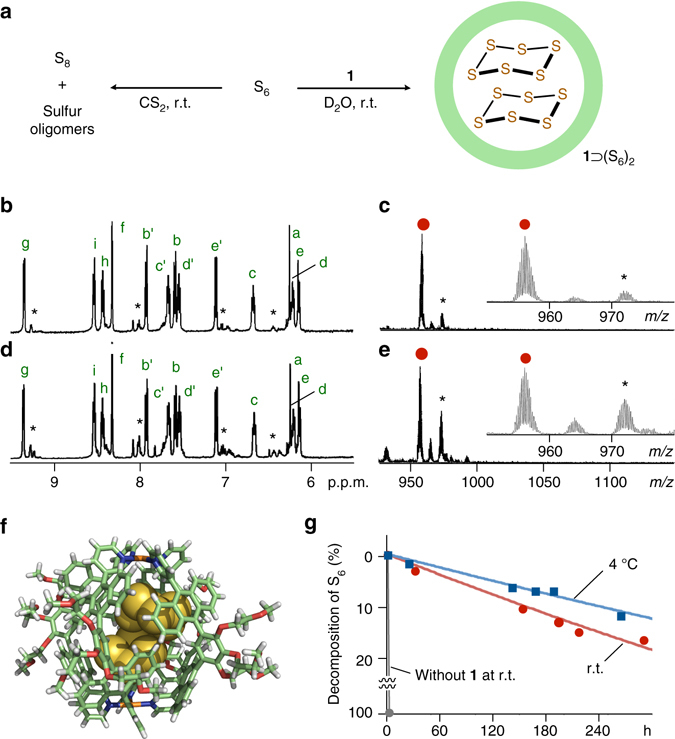
Formation and characterization of **1**⊃(S_6_)_2_ and its stability. **a** Schematic representation of the encapsulation of two S_6_ clusters within matrix **1** in D_2_O (*right*) and the decomposition of S_6_ in CS_2_ (*left*). ^1^H NMR (500 MHz, D_2_O, room temperature; *left*) and ESI-TOF MS spectra (H_2_O; *right*) of **1**⊃(S_6_)_2_ **b**, **c** before and **d**, **e** after 8 days at room temperature (*asterisks* indicate signals derived from **1**⊃(S_6_•S_8_)). **f** X-ray crystal structure of **1**⊃(S_6_)_2_: cylindrical and space-filling representation (solvents and counterions are omitted for clarity). **g** Decomposition of S_6_ (%) within matrix **1** in water and without matrix **1** in CS_2_

To investigate how capsular matrix **1** impacts the MS analysis of a metastable sulfur allotrope, we next synthesized pure *cyclo*-hexasulfur (S_6_) at low temperatures and prepared the host–guest complex^31^. In a manner similar to the encapsulation of S_8_, simple mixing hydrophobic S_6_ (13 equiv.) with **1** in D_2_O gave rise to a **1**⊃(S_6_)_2_ complex predominantly (Fig. 3a). A ^1^H NMR spectrum of the resultant solution displayed new prominent signals in the aromatic region derived from the desired product (Fig. 3b and see Supplementary Fig. 10). One set of minor signals was also found at, e.g., 9.27, 8.07, and 8.01 p.p.m., which suggests the formation of a **1**⊃(S_6_•S_8_) complex (<10%) due to the decomposition of S_6_ during the mixing. UV-visible absorption bands of **1** around 390 nm were again slightly red-shifted (Δ*λ*_max_ = +3 nm) after treatment with the S_6_ solid (see Supplementary Fig. 11). The encapsulation of two cyclic S_6_ clusters within the matrix was preliminarily confirmed by the X-ray crystallographic analysis (Fig. 3f and see Supplementary Figs 12 and 13) and yet definitely proved by the ESI-TOF MS analysis. Prominent molecular ion peaks were observed in the spectrum at *m/z* = 956.0, 1295.4, and 1974.6, derived from [**1**⊃(S_6_)_2_−*n•*NO_3_^−^]^*n*+^ species (*n* = 4, 3, and 2, respectively; Fig. 3c). The isotope patterns of the peaks fully coincide with the calculated ones (see Supplementary Fig. 14). As expected, minor MS peaks found at *m/z* = 972.0 and 1317.0 were assignable to a **1**⊃(S_6_•S_8_) species. In contrast to the host–guest complex, without the matrix, the MALDI-TOF MS analysis of free S_6_ showed only the fragment peaks (see Supplementary Fig. 15).

It is worth noting that the otherwise labile S_6_ cluster, which possesses a highly strained ring structure^4, 6, 32^, is remarkably stabilized within matrix **1** not only under MS conditions but also in an ambient solution state^33^. Free S_6_ dissolved in CS_2_ was completely decomposed into S_8_ and other oligosulfurs within ~1 h at room temperature (Fig. 3g and see Supplementary Fig. 17). In sharp contrast, time-dependent ^1^H NMR analysis revealed that 75% of the proton signals of **1**⊃(S_6_)_2_ remain unchanged after 8 days (Fig. 3d and see Supplementary Fig. 18) and the ESI-TOF MS analysis of the resultant solution indicated the generation of **1**⊃(S_6_•S_8_) and **1**⊃(S_6_•S_7_) species (Fig. 3e). The *τ*_1/2_ of the S_6_ clusters within **1** was estimated to be 770 h by ^1^H NMR studies (Fig. 3g). The decomposition is further suppressed at lower temperatures (e.g., *τ*_1/2_ = 1733 h at 4 °C). The observed, unusual stabilization of encapsulated S_6_ clusters in solution as well as under the MS conditions presumably arises from the isolation effect of the polyaromatic shell of matrix **1**.

### In situ preparation and MS analysis of a cyclic S_12_ cluster

**Fig. 4:**
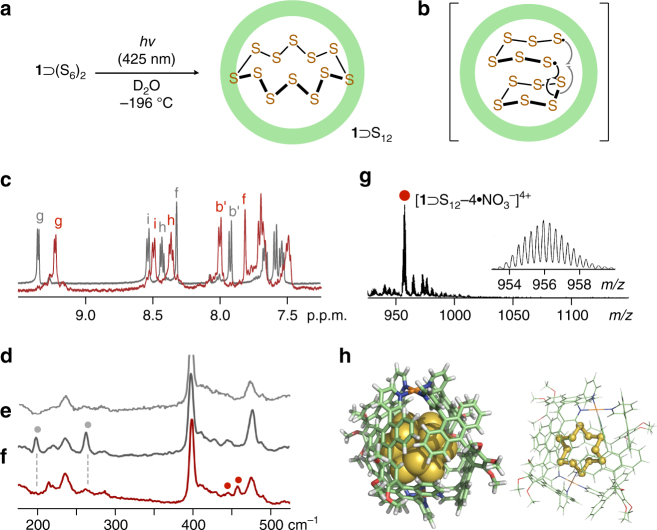
Preparation and characterization of **1**⊃S_12_. **a** Schematic representation of the transformation from two S_6_ clusters into a S_12_ cluster within matrix **1** upon light irradiation and **b** the proposed intermediate. **c** ^1^H NMR spectra (500 MHz, D_2_O, room temperature) of **1**⊃S_12_ (*red line*) and **1**⊃(S_6_)_2_ (*gray line*). Raman spectra (He−Ne laser, *λ*_ex_ = 632.8 nm, room temperature) of **d** **1**, **e** **1**⊃(S_6_)_2_, and **f** **1**⊃S_12_. **g** ESI-TOF MS spectrum (H_2_O) of **1**⊃S_12_. **h** Optimized structure of **1**ʹ⊃S_12_ (R = −OCH_3_): cylindrical and space-filling representation (*left*) and ball-and-stick representation (*right*)

It is known that sulfur clusters are photosensitive and, in most cases, rapidly decomposed into complex mixtures in solutions upon light irradiation^4–6^. Notably, selective photochemical transformation from two S_6_ clusters into a cyclic S_12_ cluster and subsequent MS analysis of the product were attained using matrix **1**. When a frozen aqueous glass of **1**⊃(S_6_)_2_ cooled at −196 °C was irradiated by LED lamps (3 W × 4, *λ*_irrd_ = 425 ± 15 nm) for 30 min, distinct shifts of the matrix signals were observed in the NMR spectrum (Figs. 4a, c and see Supplementary Fig. 19). The signal pattern is quite different from that of empty **1** and host–guest complex **1**⊃(S_8_)_2_, elucidating the formation of a new photoproduct within **1**. In addition, a huge upfield shift of inner protons *H*_f_ is indicative of the encapsulation of a bulky cluster. The product could not be extracted with CS_2_ solutions, because of most probably its steric demand. This photoreaction occurred very slowly under UV light irradiation (*λ*_irrd_ = 360 nm), probably due to the strong absorption bands (~380 nm) of the polyaromatic capsule (see Supplementary Fig. 20). A Raman spectrum of the product supported the formation of the cyclic S_12_ cluster (Figs. 4d, f and see Supplementary Fig. 21). The observed new peaks at 445 and 457 cm^−1^ are overlapped with the characteristic peaks of free S_12_ (i.e., 445 and 456 cm^−1^, respectively)^34^. On the other hand, Raman peaks derived from clusters S_6_ (e.g., 200 and 264 cm^−1^; Fig. 4e) and S_8_ (e.g., 149 and 218 cm^−1^) within **1** were virtually undetected in the spectrum. These peaks are slightly shifted (up to −4 cm^−1^) as compared with those of free S_6_ and S_8_ (see Supplementary Figs 16 and 9). Finally, ESI-TOF MS spectrum of the product exhibited intense peaks at *m/z* = 956.0 and 1295.7, assigned to 1:1 host–guest [**1**⊃S_12_−*n•*NO_3_^−^]^*n*+^ species (*n* = 4 and 3, respectively; Fig. 4g and see Supplementary Fig. 22). The MS peaks for **1** and **1**⊃(S_8_)_2_ were not observed in the spectrum. The optimized structure of **1**ʹ⊃S_12_ (R = −OCH_3_) by force-field calculations displays that the spherical S_12_ cluster with a diameter of 0.66 nm is fully insulated by the spherical polyaromatic framework of **1**ʹ (Fig. 4h and see Supplementary Fig. 23). The otherwise reactive S_12_ cluster in the capsular shell remains in more than 60% for 8 days at room temperature (see Supplementary Figs 24 and 25). Concerning the unusual clusterization in the isolated nanospace, the S_6_ cluster provides a highly strained, small ring structure, whereas the product is the next stable sulfur allotrope after S_8_. Thus, the formation of the S_12_ cluster through the generation of a biradical species (Fig. 4b), by homolytic photocleavage of the S–S bond of the encapsulated S_6_, might be a thermodynamically favorable process^32, 35^.

## Discussion

We have succeeded in the facile MS characterization of inorganic sulfur clusters S_6_, S_8_, and S_12_ by using a polyaromatic capsule as a new supramolecular matrix, whereas the clusters themselves fragment under usual MS conditions. Simple mixing cyclic S_6_ or S_8_ clusters with the capsular matrix provides stable 1:2 host–guest complexes for common ESI-TOF MS analysis. The cationic and closed polyaromatic shell of the matrix facilitates the efficient ionization of the uncharged clusters without troublesome fragmentation. In addition, the capsular matrix can be used for not only in situ synthesis but also MS analysis of a cyclic S_12_ cluster. The otherwise labile S_6_ and S_12_ clusters are also significantly stabilized within the matrix in an ambient solution state. The present functions as an analytical tool as well as a reaction vessel for sulfur clusters prompt us to discover unknown inorganic clusters by using the polyaromatic capsular matrix and its potential derivatives^36^.

## Methods

### General

NMR: Bruker ASCEND-500 (500 MHz), ESI-TOF MS: Bruker micrOTOF II, FT-IR: JASCO FT/IR-4200, X-ray: Bruker AXS D8 VENTURE/PHOTON 100 diffractometer Raman: JASCO NRS-4100 or HORIBA, Ltd LabRAM HR Evolution. Optimized structure: Materials Studio (ver. 5.5.3). Solvents and reagents: TCI Co., Ltd, Wako Pure Chemical Industries Ltd, Kanto Chemical Co., Inc., Sigma-Aldrich Co., and Cambridge Isotope Laboratories, Inc. Polyaromatic capsule **1** and sulfur cluster S_6_ (see Supplementary Methods) were synthesized according to previously reported procedures^18, 31^.

### Raman analysis

The excitation wavelength from an He-Ne laser was 632.8 nm with a power on the sample typically 22 μW. A long-working distance objective (× 50 magnification, 10 mm focal length) was used to focus the laser onto the sample. The Raman signal was collected in a back-scattering geometry.

### Synthesis of 1⊃(S_8_)_2_

Capsular matrix **1** (1.5 mg, 0.40 μmol), S_8_ (1.0 mg, 3.9 μmol), and D_2_O (0.5 ml) were added to a microtube (2 ml) and the resultant mixture was stirred at room temperature for 30 min. The quantitative formation of a **1**⊃(S_8_)_2_ complex was confirmed by NMR (see Supplementary Figs 1 and 2), X-ray crystallographic (see Supplementary Figs 3 and 4 and Supplementary Table 1), ESI-TOF MS (see Supplementary Fig. 6), and Raman (see Supplementary Fig. 9) analyses. The host–guest structure of **1**⊃(S_8_)_2_ is stable enough in water at room temperature even under high dilution conditions (5.0 μM), as confirmed by ^1^H NMR analysis (see Supplementary Fig. 7).

^1^H NMR (500 MHz, D_2_O, room temperature): *δ* 2.40 (s, 24H), 3.00 (m, 16H), 3.44 (s, 12H), 3.90 (m, 16H), 4.02 (m, 8H), 4.42 (m, 4H), 4.60 (m, 4H), 6.27 (s, 4H), 6.38 (d, *J* = 8.5 Hz, 8H), 6.80 (dd, *J* = 8.5, 7.5 Hz, 8H), 6.96 (d, *J* = 8.5 Hz, 8H), 7.16 (dd, *J* = 8.5, 7.5 Hz, 8H), 7.52 (dd, *J* = 8.5, 7.5 Hz, 8H), 7.68 (s, 8H), 7.72 (d, *J* = 8.5 Hz, 8H), 7.78 (dd, *J* = 8.5, 7.5 Hz, 8H), 8.05 (d, *J* = 8.5 Hz, 8H), 8.33 (dd, *J* = 8.0, 5.5 Hz, 8H), 8.56 (d, *J* = 8.0 Hz, 8H), 9.10 (d, *J* = 5.5 Hz, 8H). ^13^C NMR (125 MHz, D_2_O, room temperature): *δ* 57.0 (CH_3_), 58.1 (CH_3_), 70.6 (CH_2_), 71.7 (CH_2_), 72.6 (CH_2_), 72.7 (CH_2_), 124.2 (CH), 124.8 (CH), 126.2 (CH), 126.5 (CH), 127.0 (CH), 127.2 (CH), 127.3 (CH), 127.7 (C_*q*_), 128.1 (C_*q*_), 128.4 (CH), 129.3 (C_*q*_), 129.4 (C_*q*_), 129.5 (C_*q*_), 129.8 (CH), 134.9 (C_*q*_), 138.4 (C_*q*_), 145.3 (CH), 145.6 (C_*q*_), 151.5 (C_*q*_), 151.7 (CH), 152.1 (CH). Raman (*λ*_ex_ = 632.8 nm, 22 μW, cm^−1^): 149, 218, 238, 400, 478. ESI-TOF MS (H_2_O): *m/z* 2038.6 [**1**⊃(S_8_)_2_−2•NO_3_^−^]^2+^, 1338.1 [**1**⊃(S_8_)_2_−3•NO_3_^−^]^3+^, 988.0 [**1**⊃(S_8_)_2_−4•NO_3_^−^]^4+^.

### Synthesis of 1⊃(S_6_)_2_

Capsular matrix **1** (1.5 mg, 0.40 μmol), S_6_ (1.0 mg, 5.1 μmol), and D_2_O (0.5 ml) were added to a microtube (2 ml). The mixture was stirred at room temperature for 15 min. The selective formation of a **1**⊃(S_6_)_2_ complex (>90% yield) was confirmed by NMR (see Supplementary Fig. 10), UV-visible (see Supplementary Fig. 11), X-ray crystallographic (see Supplementary Figs 12 and 13 and Supplementary Table 2), ESI-TOF MS (see Supplementary Fig. 14), and Raman (see Supplementary Fig. 16) analyses.

^1^H NMR (500 MHz, D_2_O, room temperature): *δ* 2.39 (s, 24H), 3.00 (m, 16H), 3.43 (s, 12H), 3.82 (m, 8H), 3.89 (m, 8H), 3.96 (m, 8H), 4.39 (m, 4H), 4.59 (m, 4H), 6.14 (d, *J* = 8.5 Hz, 8H), 6.21 (dd, *J* = 8.5, 7.5 Hz, 8H), 6.25 (s, 4H), 6.67 (dd, *J* = 8.5, 7.5 Hz, 8H), 7.11 (d, *J* = 8.5 Hz, 8H), 7.54 (dd, *J* = 8.5, 7.5 Hz, 8H), 7.59 (d, *J* = 8.5 Hz, 8H), 7.66 (dd, *J* = 8.5, 7.5 Hz, 8H), 7.92 (d, *J* = 8.5 Hz, 8H), 8.32 (s, 8H), 8.43 (dd, *J* = 8.0, 6.0 Hz, 8H), 8.53 (d, *J* = 8.0 Hz, 8H), 9.35 (d, *J* = 6.0 Hz, 8H). Raman (*λ*_ex_ = 632.8 nm, 22 μW, cm^−1^): 200, 238, 264, 398, 478. ESI-TOF MS (H_2_O): *m/z* 1974.6 [**1**⊃(S_6_)_2_−2•NO_3_^−^]^2+^, 1295.4 [**1**⊃(S_6_)_2_−3•NO_3_^–^]^3+^, 956.0 [**1**⊃(S_6_)_2_−4•NO_3_^–^]^4+^.

### Formation of 1⊃S_12_

An aqueous solution of **1**⊃(S_6_)_2_ (0.8 mM, 0.5 ml) was irradiated with Relyon LED lamps (3 W × 4: *λ*_irrd_ = 425 ± 15 nm) for 30 min at −196 °C (under liquid N_2_). The selective formation of a **1**⊃S_12_ complex was confirmed by ^1^H NMR (see Supplementary Fig. 19), UV-visible (see Supplementary Fig. 20), Raman (see Supplementary Fig. 21), and ESI-TOF MS (see Supplementary Fig. 22) analyses. On the other hand, light irradiation (3 W × 2) of **1**⊃(S_6_)_2_ in D_2_O for 2 h at room temperature gave rise to complex mixtures.

^1^H NMR (500 MHz, D_2_O, room temperature): *δ* 2.39 (s, 24H), 3.02 (m, 16H), 3.45 (s, 12H), 3.92 (m, 16H), 3.99 (m, 8H), 4.44 (m, 4H), 4.61 (m, 4H), 6.15 (d, *J* = 8.5 Hz, 8H), 6.36 (dd, *J* = 8.5, 7.0 Hz, 8H), 6.49 (s, 4H), 6.92 (dd, *J* = 8.5, 7.0 Hz, 8H), 7.01 (d, *J* = 8.5 Hz, 8H), 7.49 (dd, *J* = 8.5, 7.0 Hz, 8H), 7.72-7.69 (m, 16H), 7.82 (s, 8H), 8.00 (d, *J* = 8.5 Hz, 8H), 8.36 (dd, *J* = 7.5, 5.5 Hz, 8H), 8.49 (d, *J* = 7.5 Hz, 8H), 9.22 (d, *J* = 5.5 Hz, 8H). Raman (*λ*_ex_ = 632.8 nm, 22 μW, cm^−1^): 180, 216, 238, 263, 288, 398, 410, 445, 457, 475, 490. ESI-TOF MS (H_2_O): *m/z* 1974.0 [**1**⊃S_12_ – 2•NO_3_^−^]^2+^, 1295.7 [**1**⊃S_12_ – 3•NO_3_^−^]^3+^, 956.0 [**1**⊃S_12_ – 4•NO_3_^−^]^4+^.

### Data availability

The authors declare that the data supporting the findings of this study are available within the Supplementary Information files and from the corresponding author upon reasonable request. CCDC-1509515 and CCDC-1525707 contain the supplementary crystallographic data for the structures reported in this article. These data can be obtained free of charge from The Cambridge Crystallographic Data Centre (CCDC) via www.ccdc.cam.ac.uk/data_request/cif.

## Electronic supplementary material

Supplementary Information

Peer Review File-New

Supplementary Data 1

Supplementary Data 2

Supplementary Data 3

Supplementary Data 4
