# Supplementary material for: Exact mass analysis of sulfur clusters upon encapsulation by a polyaromatic capsular matrix
Source: Nat Commun. 2017 Sep 29;8:749. doi: 10.1038/s41467-017-00605-5 (PMC5622087; doi:10.1038/s41467-017-00605-5)
Supplement: Supplementary file 1 — Supplementary Information [file 41467_2017_605_MOESM1_ESM.pdf]

Description of Supplementary Files

File Name: Supplementary Information
Description: Supplementary Figures, Supplementary Tables, Supplementary Methods, Supplementary References.

File Name: Supplementary Data 1
Description: CIF_SM253

File Name: Supplementary Data 2
Description: CIF_SM321

File Name: Supplementary Data 3
Description: checkcif_SM253

File Name: Supplementary Data 4
Description: checkcif_SM321

File Name: Peer Review File

Supplementary Figures

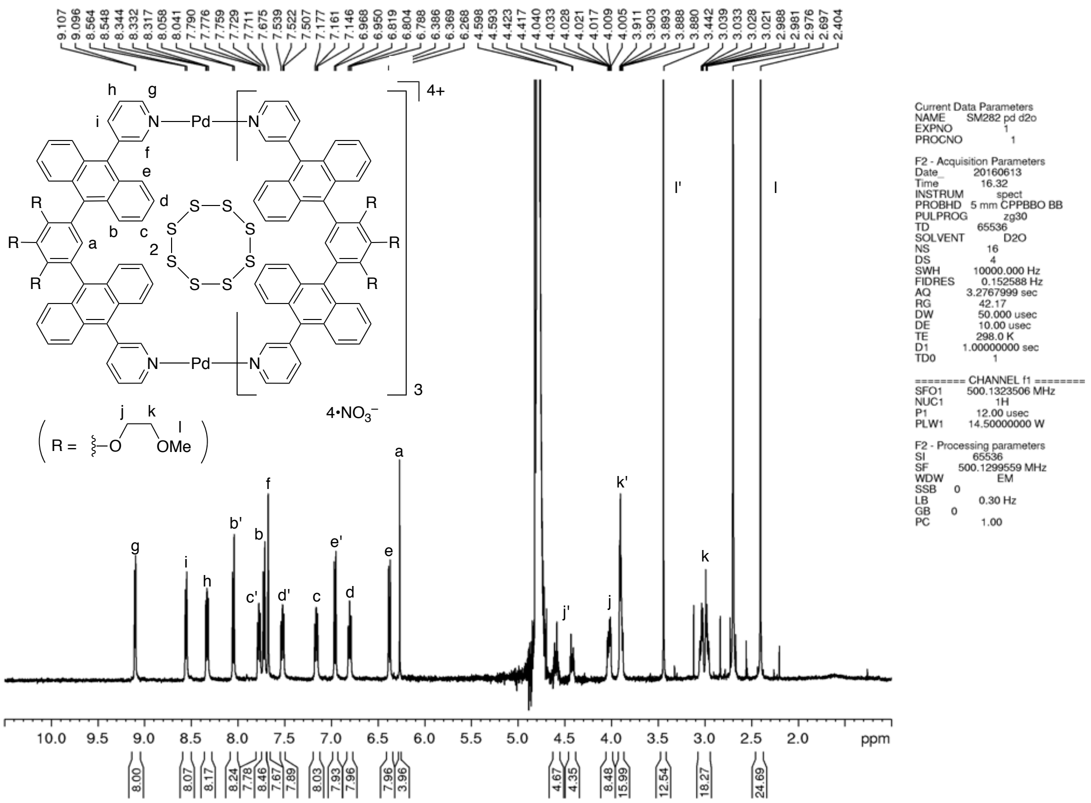

Supplementary Figure 1 | ^{1}H NMR spectrum (500 MHz, D_2O, r.t.) of **1**⊃$(S_8)_2$.

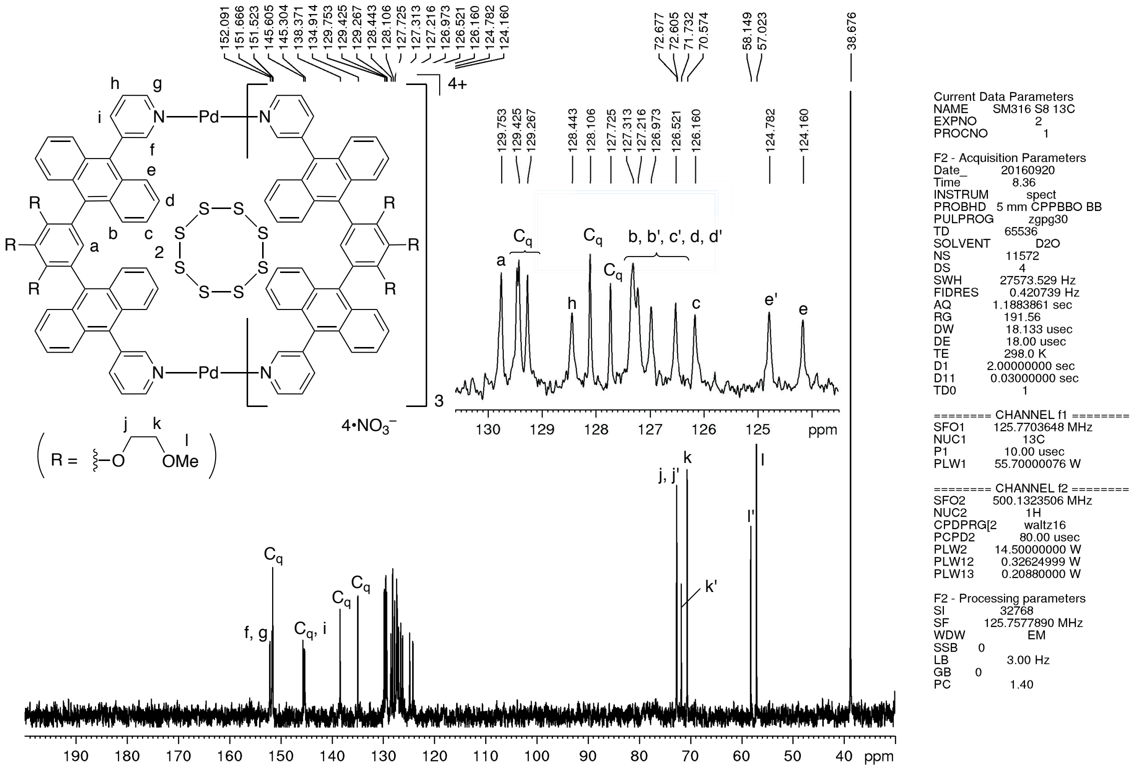

Supplementary Figure 2 | ^{13}C NMR spectrum (125 MHz, D_2O, r.t.) of **1**⊃$(S_8)_2$.

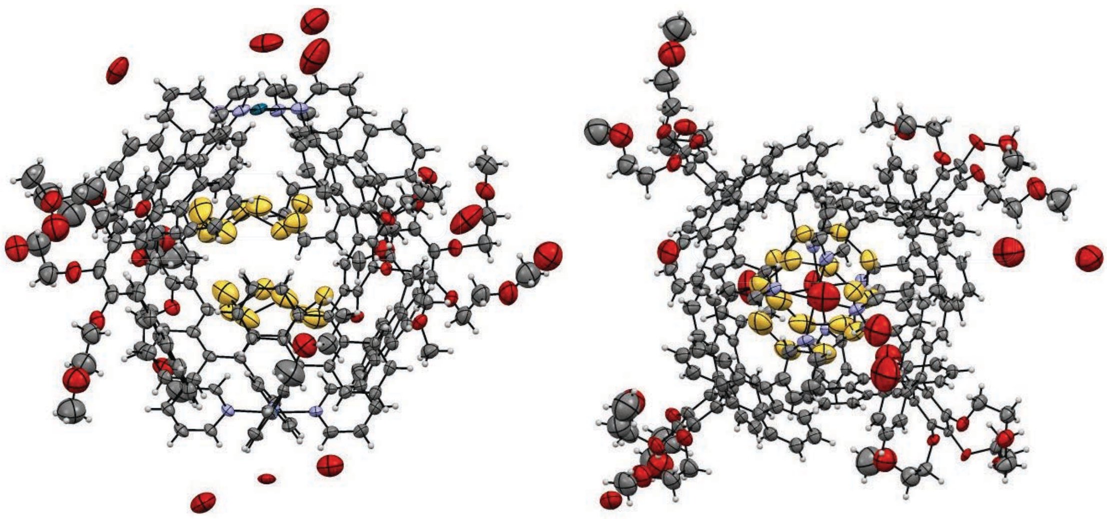

Supplementary Figure 3 | ORTEP drawing of **1**⊃$(S_8)_2$ (side and top views). Disordered counterions and solvent molecules are omitted for clarity.

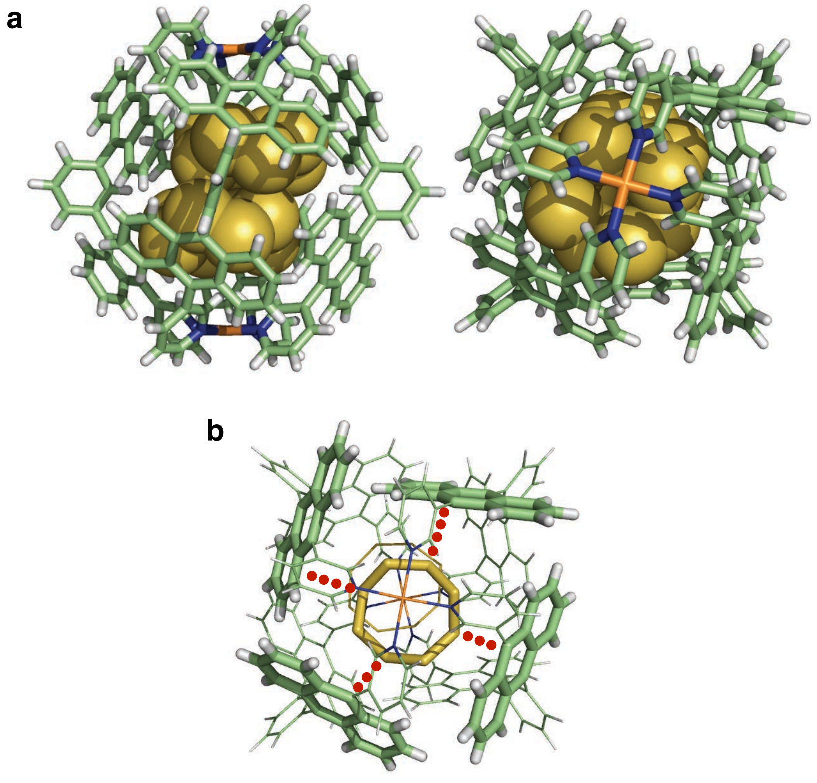

Supplementary Figure 4 | (a) Cylindrical (for **1**) and space-filling (for S_8) representation of the crystal structure (main frameworks) of **1**⊃$(S_8)_2$ (side and top views). (b) Selected S-π distance (≤3.6 Å) between one of the S_8 clusters and the anthracene panels within **1**⊃$(S_8)_2$.

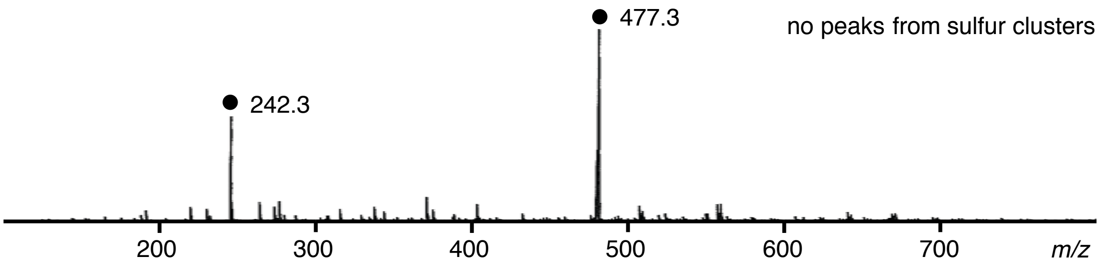

Supplementary Figure 5 | ESI-TOF MS spectrum (CH_2Cl_2) of S_8.

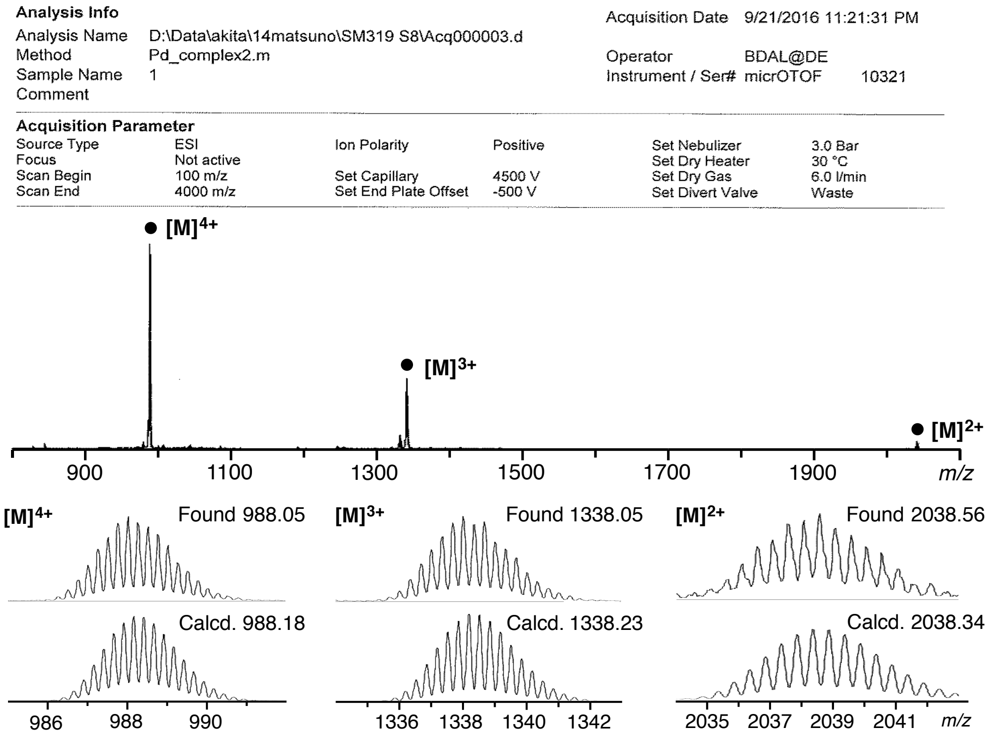

Supplementary Figure 6 | ESI-TOF MS spectrum (H_2O) of $\mathbf{1}\supset(S_8)_2$.

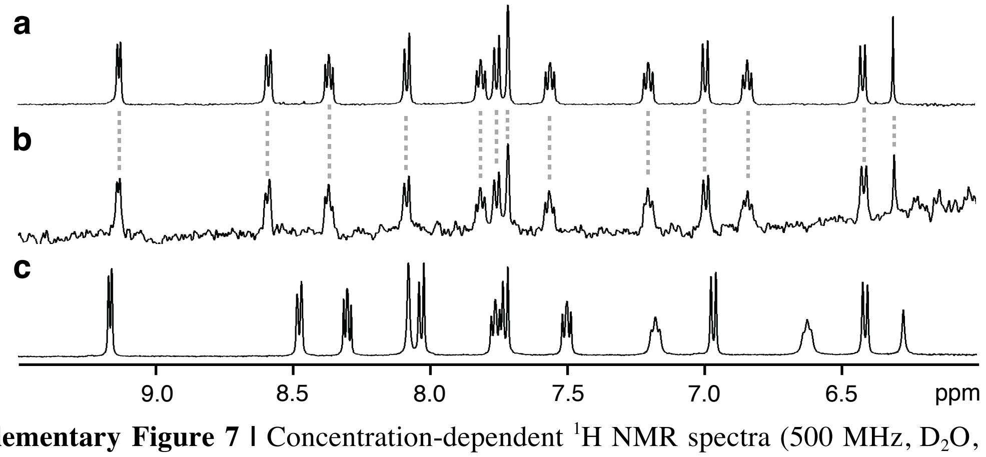

Supplementary Figure 7 | Concentration-dependent ^{1}H NMR spectra (500 MHz, D_2O, r.t.) of $\mathbf{1}\supset(S_8)_2$ at (a) 0.8 mM, (b) 5.0 μM, and of (d) empty capsule **1**.

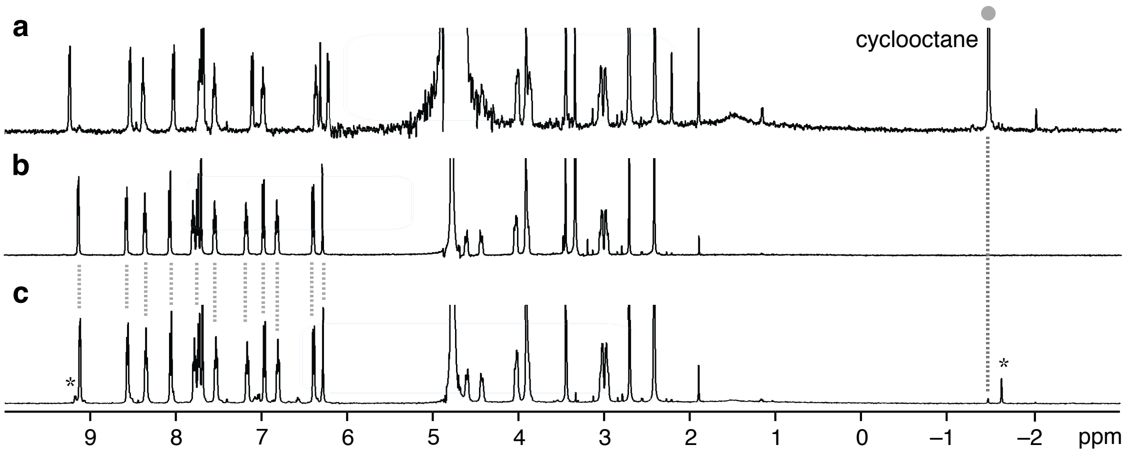

Supplementary Figure 8 | ^{1}H NMR spectra (500 MHz, D_2O, r.t.) of (a) **1**⊃(cyclooctane)$_2$, (b) **1**⊃(S_8)$_2$, and (c) **1**⊃(cyclooctane)$_2$ after mixing with S_8 at r.t. for 3 h. ∗: **1**⊃(S_8•cyclooctane).

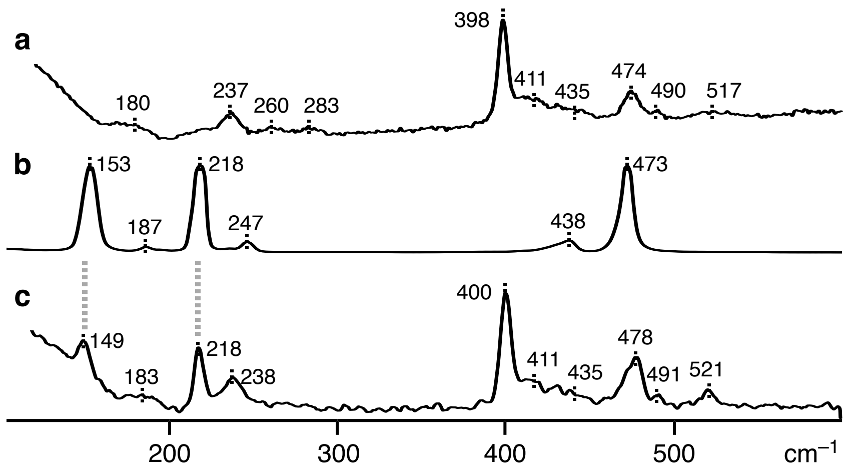

Supplementary Figure 9 | Raman spectra (λ_{ex} = 632.8 nm, solid, r.t.) of (a) **1**, (b) S_8, and (c) **1**⊃(S_8)$_2$.

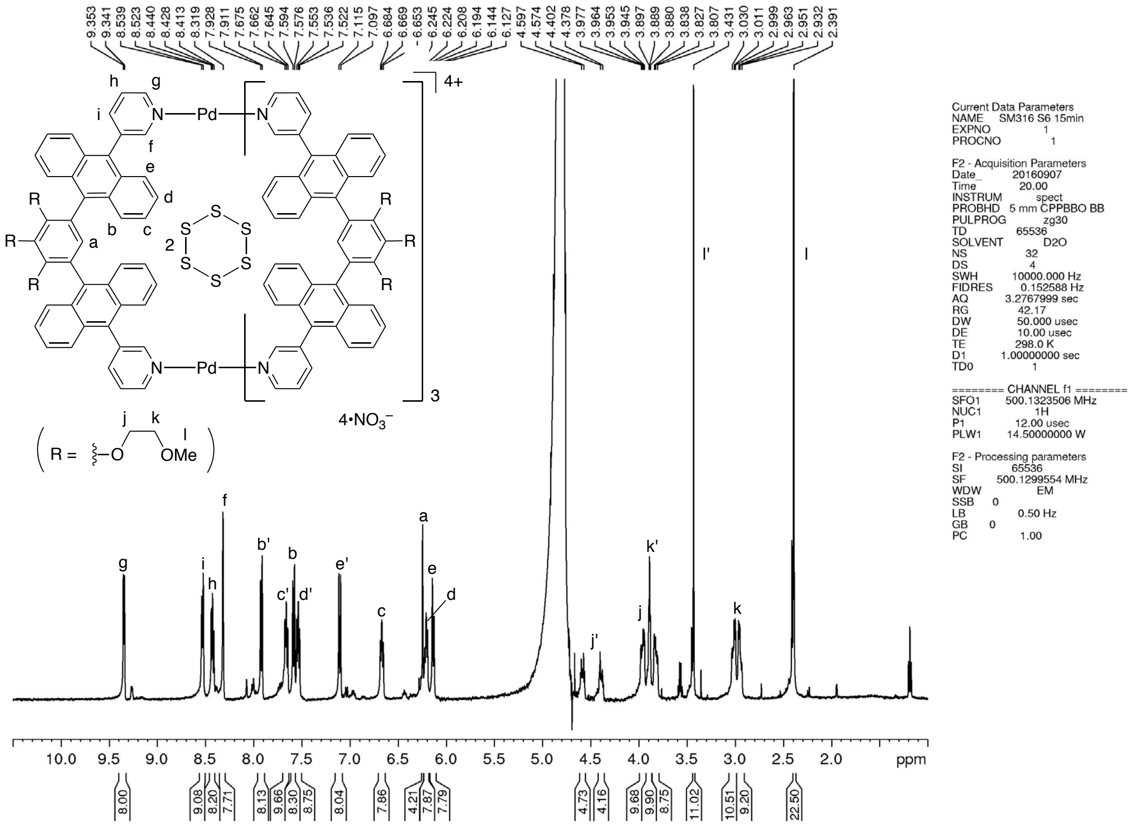

Supplementary Figure 10 | ^{1}H NMR spectrum (500 MHz, D_2O, r.t.) of **1**⊃$(S_6)_2$.

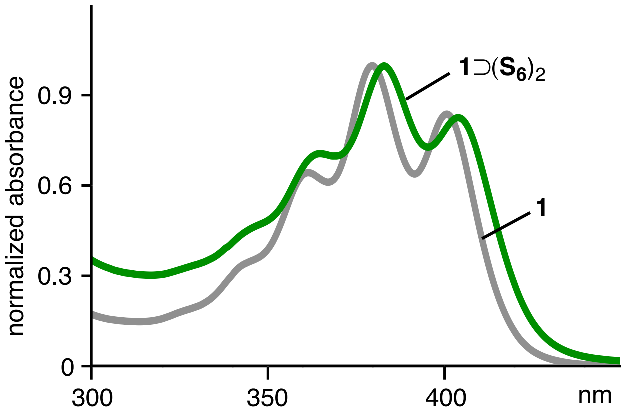

Supplementary Figure 11 | UV-visible spectra (H_2O, r.t.) of **1** and **1**⊃$(S_6)_2$.

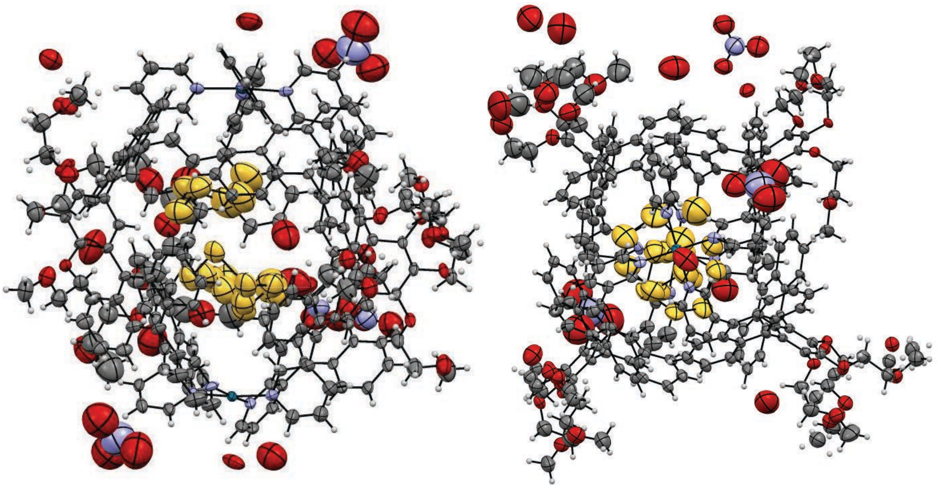

Supplementary Figure 12 | ORTEP drawing of **1**⊃$(S_6)_2$ (side and top views).

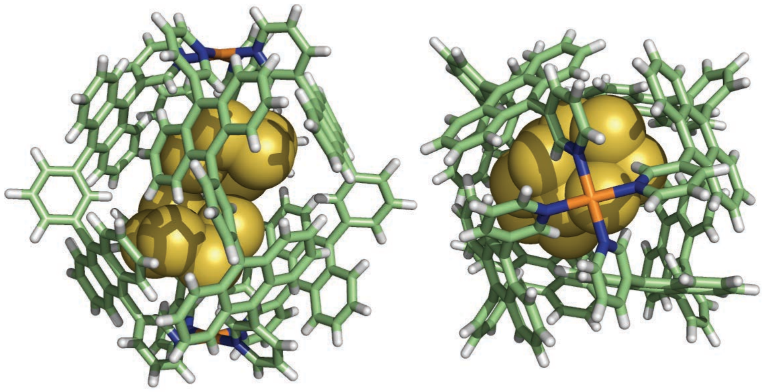

Supplementary Figure 13 | Cylindrical (for **1**) and space-filling (for S_6) representations of the crystal structure (main frameworks) of **1**⊃$(S_6)_2$ (side and top views). Substituents, solvents, disordered guests and counterions are omitted for clarity.

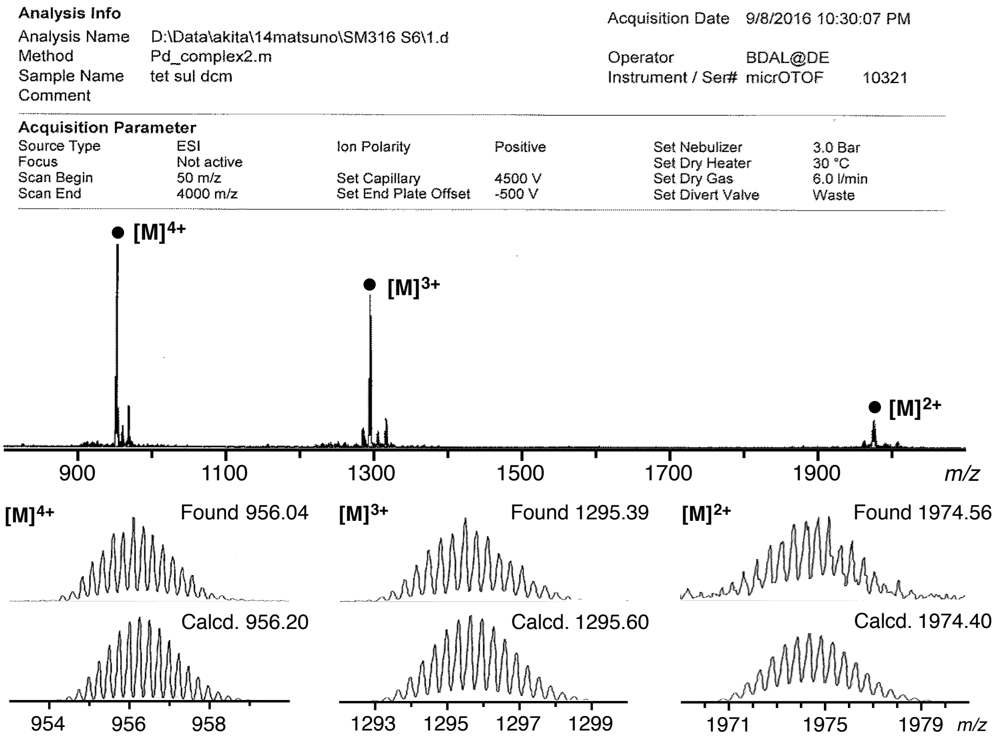

Supplementary Figure 14 | ESI-TOF MS spectrum (H_2O) of **1**$\supset(S_6)_2$.

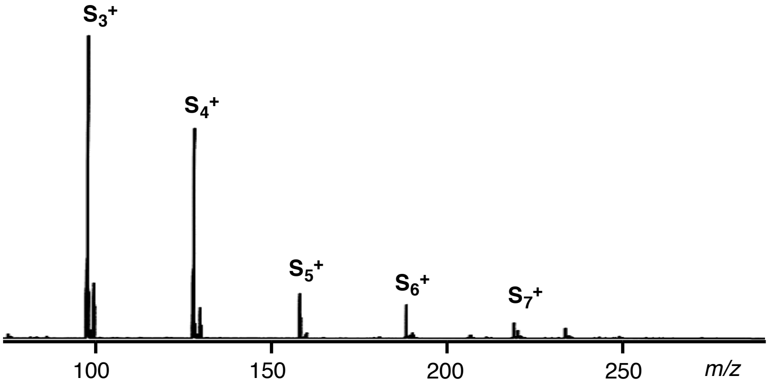

Supplementary Figure 15 | MALDI-TOF MS spectrum of S_6.

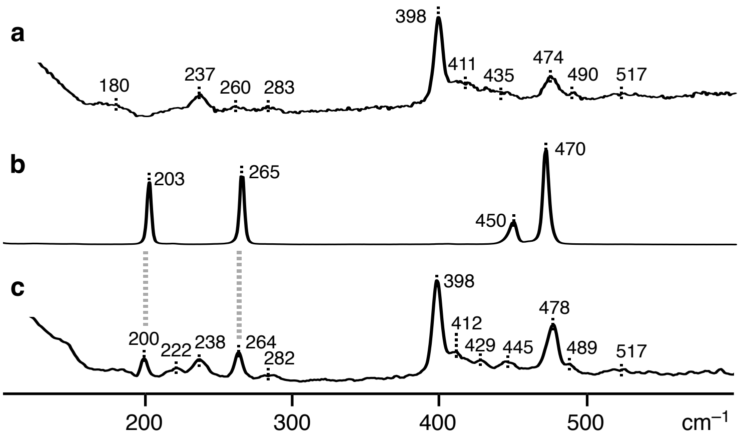

Supplementary Figure 16 | Raman spectra (λ_{ex} = 632.8 nm, solid, r.t.) of (a) **1**, (b) S_6, and (c) **1**$\supset(S_6)_2$.

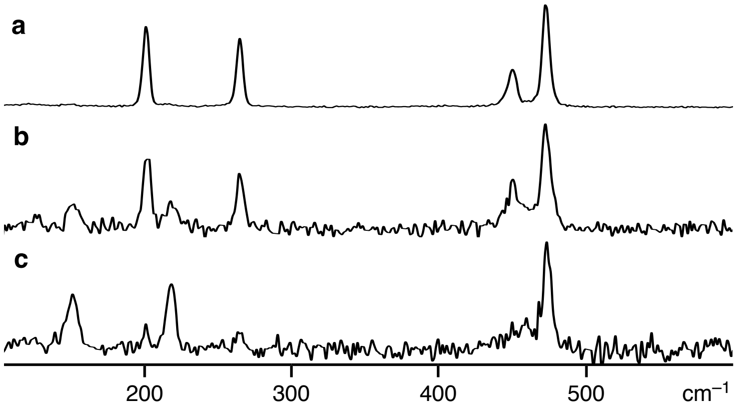

Supplementary Figure 17 | Raman spectra (λ_{ex} = 632.8 nm, solid, r.t.) of S_6 in CS_2 under ambient conditions (air and room light at r.t.) after (a) 10, (b) 30, and (c) 60 min.

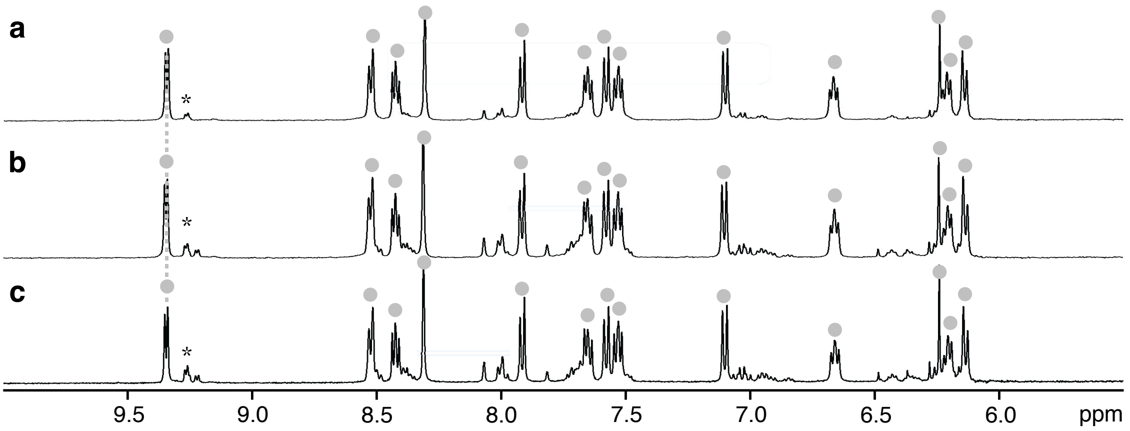

Supplementary Figure 18 | ^{1}H NMR spectra (500 MHz, D_2O, r.t.) of **1**$\supset(S_6)_2$ (a) before and (b) after 8 d under ambient conditions. ^{1}H NMR spectra (500 MHz, D_2O, r.t.) of **1**$\supset(S_6)_2$ after 8 d without (c) O_2. Gray circle: **1**$\supset(S_6)_2$ and ∗: **1**$\supset(S_6{\bullet}S_8)$.

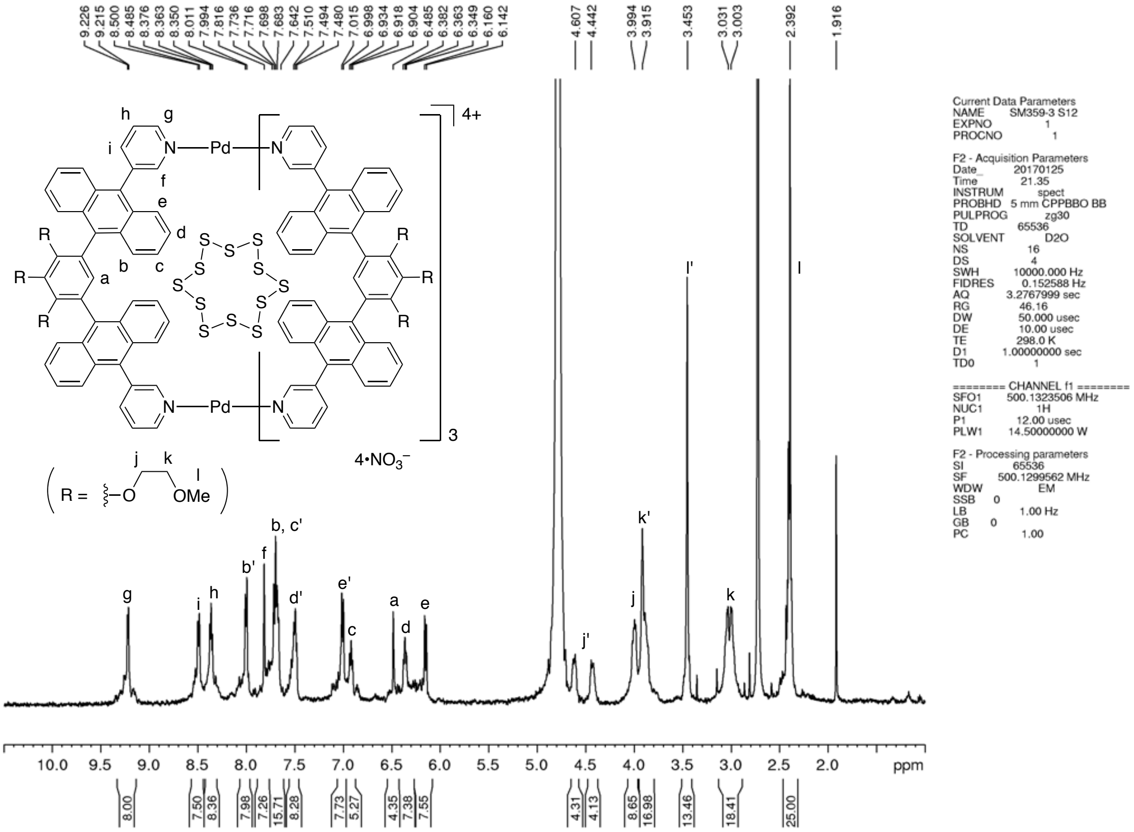

Supplementary Figure 19 | ^{1}H NMR spectrum (500 MHz, D_2O, r.t.) of **1**⊃S_{12}.

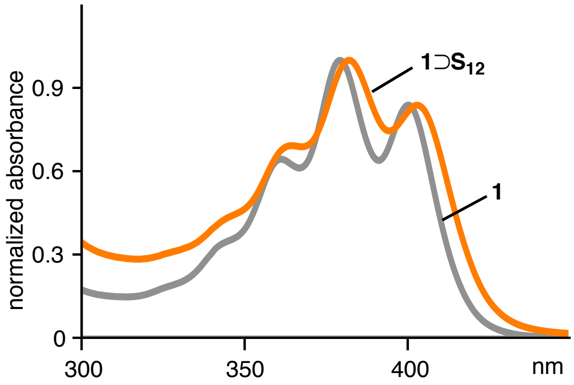

Supplementary Figure 20 | UV-visible spectra (H_2O, r.t.) of **1** and **1**⊃S_{12}.

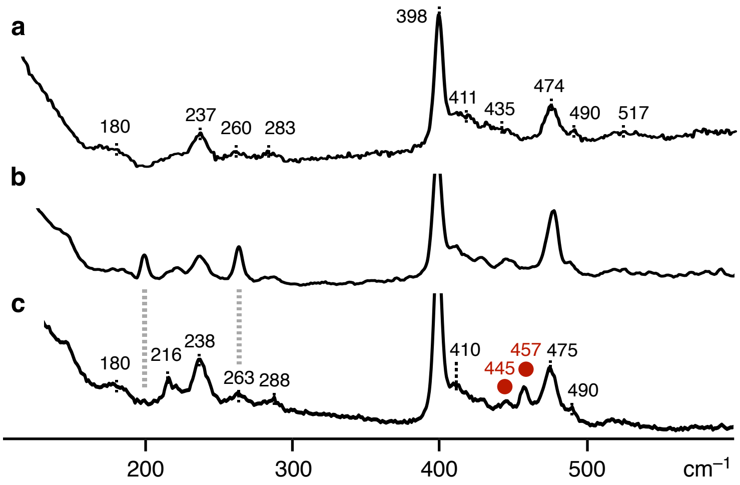

Supplementary Figure 21 | Raman spectra (λ_{ex} = 632.8 nm, solid, r.t.) of (a) **1**, (b) **1**$\supset(S_6)_2$, and (c) **1**$\supset S_{12}$.

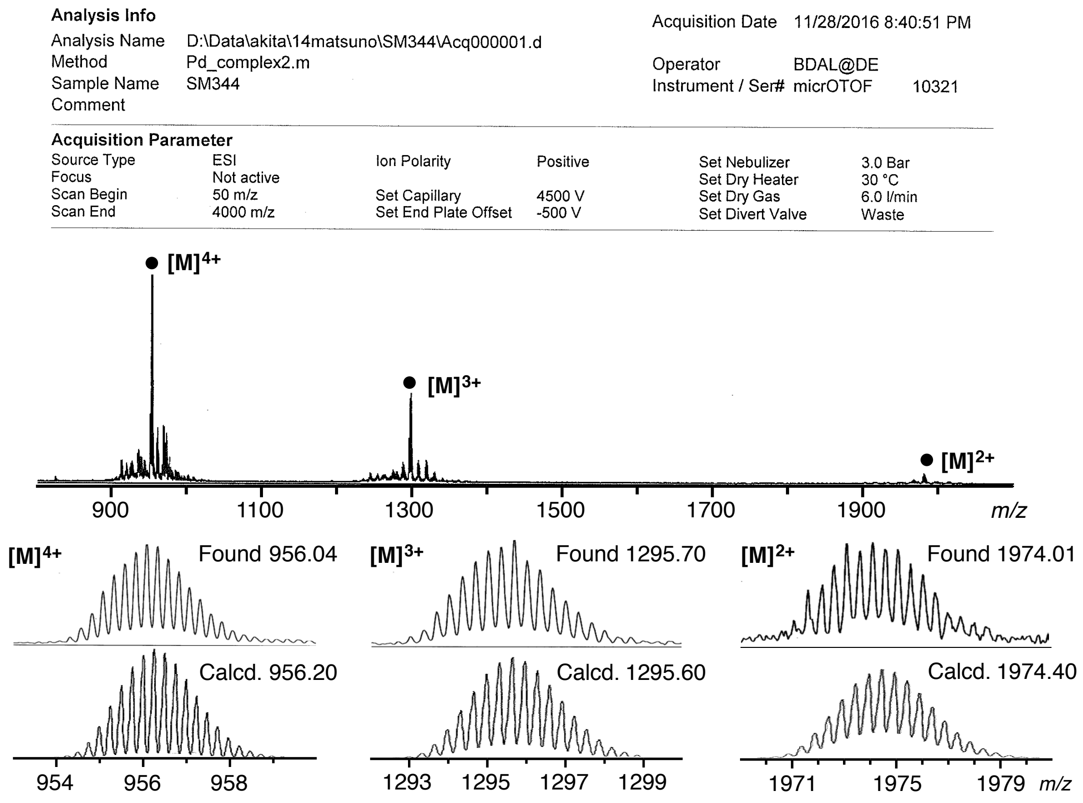

Supplementary Figure 22 | ESI-TOF MS spectrum (H_2O) of **1**$\supset S_{12}$.

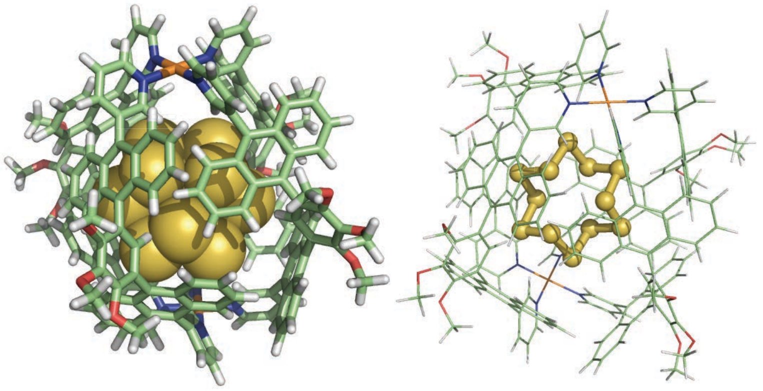

Supplementary Figure 23 | Optimized structure of **1’**⊃S_{12} (R = -OCH_3).

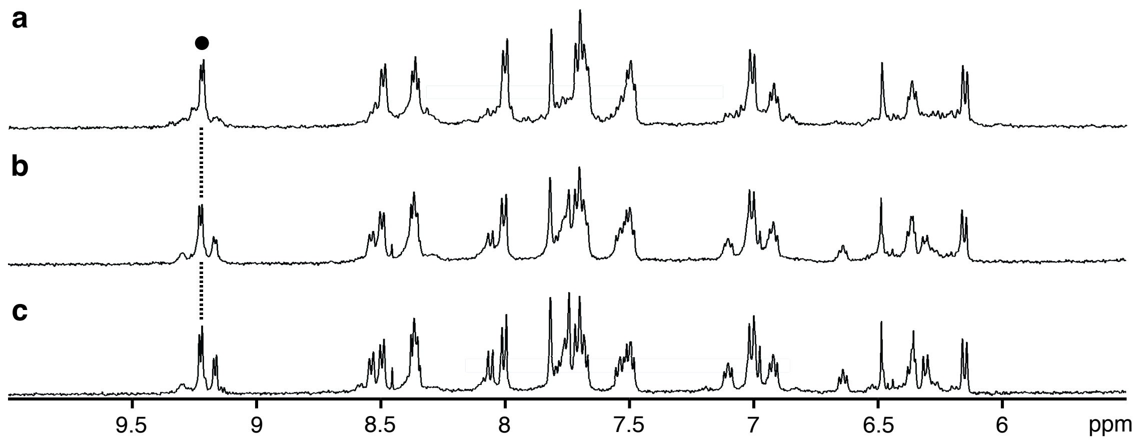

Supplementary Figure 24 | ^{1}H NMR spectra (500 MHz, D_2O, r.t.) of **1**⊃S_{12} (a) before and (b) 8 d and (c) 17 d under ambient conditions.

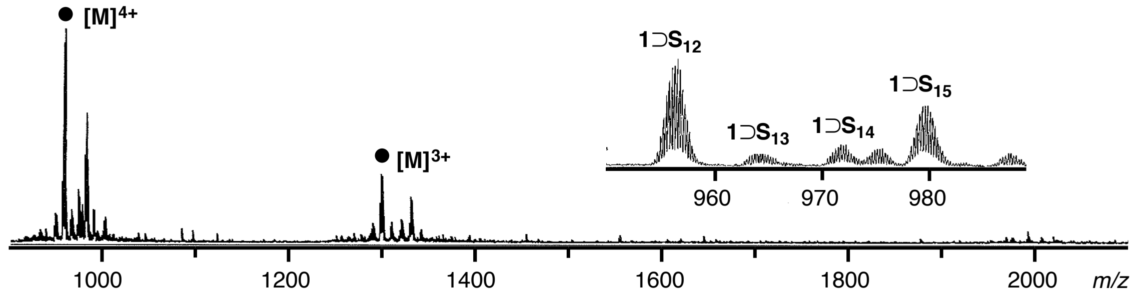

Supplementary Figure 25 | ESI-TOF MS spectrum (H_2O) of **1**⊃S_{12} after 17 d under ambient conditions.

Supplementary Tables

Supplementary Table 1 | Crystal data and structure refinement for **1**⊃$(S_8)_2$.

Identification code	SM253	
Empirical Formula	$C_{212}H_{181}N_8O_{34}Pd_2S_{16}$	
Formula Weight	4110.40	
Temperature	273 K	
Wavelength	1.54178 Å	
Crystal System	monoclinic	
Space Group	P 2_1/n	
Unit cell dimensions	a = 21.7593(11) Å	α = 90°
	b = 33.6872(16) Å	β = 92.856(3)°
	c = 28.3246(14) Å	γ = 90°
Volume	20736.4(18) $Å^3$	
Z value	4	
Density (calculated)	1.317 Mg/m^3	
Absorption coefficient	3.488 mm^{-1}	
F(000)	8516	
Crystal size	0.110 x 0.120 x 0.130 mm^3	
Theta range for data collection	2.50 to 66.60°	
Index ranges	-25<=h<=25, -37<=k<=40, -33<=l<=33	
Reflections collected	151419	
Independent reflections	36442 [R(int) = 0.1531]	
Completeness to theta = 67.596°	99.4 %	
Absorption correction	Multi-scan	
Max. and min. transmission	0.7000 and 0.6600	
Refinement method	Full-matrix least-squares on F^2	
Data / restraints / parameters	36442 / 3712 / 2449	
Goodness-of-fit on F^2	1.334	
Final R indices [I>2sigma(I)]	R_1 = 0.1355, wR_2 = 0.3575	
R indices (all data)	R_1 = 0.1955, wR_2 = 0.4022	
Largest diff. peak and hole	3.141 and –1.164 $eÅ^{-3}$	

The supplementary crystallographic data (CCDC 1509515) can be obtained free of charge from the Cambridge Crystallographic Data Centre. Disordered solvent molecules were removed by SQUEEZE program and the result was attached to the CIF file[1-4].

Supplementary Table 2 | Crystal data and structure refinement for **1**⊃$(S_6)_2$.

Identification code	SM321	
Empirical Formula	$C_{212}H_{184}N_{12}O_{46}Pd_2S_{12}$	
Formula Weight	4233.22	
Temperature	90(2) K	
Wavelength	0.71073 Å	
Crystal System	triclinic	
Space Group	P-1	
Unit cell dimensions	a = 21.297(2) Å	α = 70.0170(10)°
	b = 21.480(2) Å	β = 69.0620(10)°
	c = 27.276(3) Å	γ = 61.4680(10)°
Volume	10013.2(17) $Å^3$	
Z value	2	
Density (calculated)	1.404 Mg/m^3	
Absorption coefficient	0.387 mm^{-1}	
F(000)	4384	
Crystal size	0.040 x 0.060 x 0.100 mm^3	
Theta range for data collection	1.221 to 22.464°	
Index ranges	-22<=h<=16, -23<=k<=17, -29<=l<=26	
Reflections collected	38446	
Independent reflections	25841 [R(int) = 0.0661]	
Completeness to theta = 22.464°	99.1 %	
Absorption correction	Multi-scan	
Max. and min. transmission	0.9850 and 0.9620	
Refinement method	Full-matrix least-squares on F^2	
Data / restraints / parameters	25841 / 4572 / 2765	
Goodness-of-fit on F^2	1.060	
Final R indices [I>2sigma(I)]	R_1 = 0.1346, wR_2 = 0.3689	
R indices (all data)	R_1 = 0.1988, wR_2 = 0.4282	
Largest diff. peak and hole	2.813 and –1.393 $eÅ^{-3}$	

The supplementary crystallographic data (CCDC 1525707) can be obtained free of charge from the Cambridge Crystallographic Data Centre.

Supplementary Methods

Refinement details of X-ray crystallographic analysis of 1⊃$(S_8)_2$

The crystal structure of host-guest complex **1**⊃$(S_8)_2$ was solved using SHELXT (Sheldrick, 2014) and then refined with SHELXL-2014 (Sheldrick, 2014)[1,2]. Carbon-bound hydrogen atoms were included in idealized positions and refined using a riding model. Disorder atoms were modelled using standard crystallographic methods including constraints, restraints, and rigid bodies. The contribution of the electron density associated with greatly disordered counterions and solvent molecules, which could not be modelled with discrete atomic positions, were handled using the SQUEEZE routine in PLATON[3,4]. The crystal was susceptible to solvent loss and weakly diffracting despite long exposure times. In the checkCIF, several error messages (level B) arise from disordered oxygen atoms derived from solvent (water) molecules. Other messages (level B: high wR_2 value) are due to weak diffraction of the obtained crystal. Nevertheless, the quality of the data is more than sufficient to establish the connectivity of the capsule and cyclic S_8 structures.

Competitive binding experiment of S_8 and cyclooctane

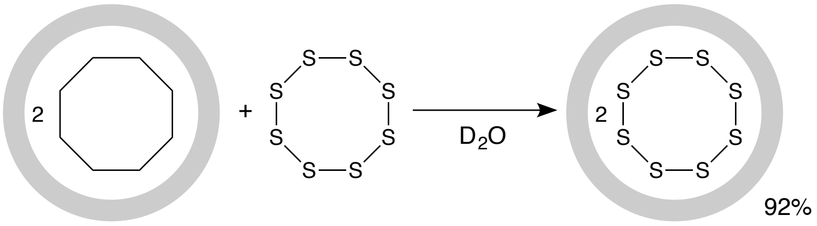

Capsule **1** (1.5 mg, 0.40 μmol), cyclooctane (1.0 mg, 8.8 μmol), and D_2O (0.5 mL) were added to a micro tube (2 mL). The mixture was stirred at room temperature for 30 min. The formation of a **1**⊃$(\text{cyclooctane})_2$ complex was confirmed by ^{1}H NMR analysis. Solid S_8 (2.3 mg, 8.8 μmol) was added to the solution. After the mixture was stirred at room temperature for 3 h, the selective formation of a **1**⊃$(S_8)_2$ complex (92%) was confirmed by ^{1}H NMR analysis (Supplementary Figure 8).

Synthesis of S_6

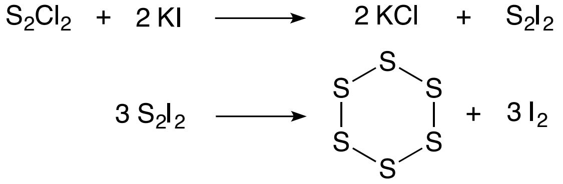

A CS_2 solution (100 mL) of S_2Cl_2 (12.1 g, 89.6 mmol) and a H_2O solution (100 mL) of KI (40.2 g, 242 mmol) were added to a 200 mL glass flask. After the mixture was stirred at room temperature for 30 min, the CS_2 layer was collected. A saturated H_2O solution (100 mL) of $Na_2S_2O_3$ was added to the separated CS_2 layer. After the mixture was stirred at room temperature for 10 min, the resultant CS_2 layer was separated and evaporated under reduced pressure. CS_2 (25 mL) was added to the obtained solution. *n*-Pentane (25 mL) was added to the mixture to give a yellow solid. Recrystallization of the yellow solid in four times from the CS_2 (15 mL) solution at –78 ºC afforded S_6 (234 mg, 14%) as an orange needle crystal.

Raman (λ_{ex} = 632.8 nm, 22 μW, cm^{-1}): 203, 265, 450, 470.

Refinement details of X-ray crystallographic analysis of 1⊃$(S_6)_2$

The crystal structure of host-guest complex **1**⊃$(S_6)_2$ was solved using SHELXT (Sheldrick, 2014) and then refined with SHELXL-2014 (Sheldrick, 2014)[1,2]. Carbon-bound hydrogen atoms were included in idealized positions and refined using a riding model. Disorder atoms were modelled using standard crystallographic methods including constraints, restraints, and rigid bodies. The occupancy factors of heavily disordered, one S_6 molecule are as follows: 68% (S1G to S6G) and 32% (S13G to S18G). The crystal was very susceptible to solvent loss and weakly diffracting despite long exposure times. Therefore, two error messages (level A: the value of sine(theta_max)/wavelength and level B: high wR_2 value) in the checkCIF arises from these crystal properties. Other messages (level B) are due to disordered oxygen atoms derived from solvent (water) molecules. Nevertheless, the quality of the data is more than sufficient to establish the connectivity of the capsule and cyclic S_6 structures. The crystal structure is fully consistent with the NMR and ESI-TOF MS data of **1**⊃$(S_6)_2$. One of the two S_6 clusters is heavily disordered in the large cavity of capsular matrix **1**.

Whereas the quality of the crystal data is not enough to discuss the structural details of the clusters, the result revealed their full accommodation within **1**.

Stability of S_6 within capsule 1

To investigate the stability of S_6 within capsule **1**, the D_2O solutions of **1**$\supset(S_6)_2$ were monitored by ^{1}H NMR and ESI-TOF MS analyses under two conditions: (i) under air and room light at room temperature and (ii) under N_2 and room light at room temperature.

Stability of S_{12} within capsule 1

To investigate the stability of S_{12} within capsule **1**, a D_2O solution of **1**$\supset S_{12}$ was monitored by ^{1}H NMR and ESI-TOF MS analyses under ambient conditions (room temperature, room light, air).

Supplementary References

1. Sheldrick, G. M. A short history of *SHELX*. *Acta Cryst. A* **64**, 112–122 (2008).
2. Sheldrick, G. M. SHELXT: Integrating space group determination and structure solution. *Acta Cryst. A* **70**, C1437 (2014).
3. Vandersluis, P. & Spek, L. A. BYPASS: an effective method for the refinement of crystal structures containing disordered solvent regions. *Acta Cryst. A* **46**, 194–201 (1990).
4. Spek, A. L. Structure validation in chemical crystallography. *Acta Cryst. D* **65**, 148–155 (2009).
