# Supplementary material for: Exact mass analysis of sulfur clusters upon encapsulation by a polyaromatic capsular matrix
Source: Nat Commun. 2017 Sep 29;8:749. doi: 10.1038/s41467-017-00605-5 (PMC5622087; doi:10.1038/s41467-017-00605-5)

checkCIF/PLATON report

You have not supplied any structure factors. As a result the full set of tests cannot be run.

THIS REPORT IS FOR GUIDANCE ONLY. IF USED AS PART OF A REVIEW PROCEDURE FOR PUBLICATION, IT SHOULD NOT REPLACE THE EXPERTISE OF AN EXPERIENCED CRYSTALLOGRAPHIC REFEREE.

No syntax errors found.   CIF dictionary   Interpreting this report

Datablock: 3_ftu1_s_sq

```
Bond precision:  C-C = 0.0152 A          Wavelength=1.54178

Cell:           a=21.7593(11)    b=33.6872(16)     c=28.3246(14)
                alpha=90         beta=92.856(3)    gamma=90
Temperature:    273 K
```

	Calculated	Reported
Volume	20736.4(18)	20736.4(18)
Space group	P 21/n	P 21/n
Hall group	-P 2yn	-P 2yn
Moiety formula	C212 H181 N8 O24 Pd2, 2(S8), 10(O)	?
Sum formula	C212 H181 N8 O34 Pd2 S16	C212 H181 N8 O34 Pd2 S16
Mr	4110.42	4110.40
Dx,g cm-3	1.317	1.317
Z	4	4
Mu (mm-1)	3.488	3.488
F000	8516.0	8516.0
F000’	8560.72	
h,k,lmax	25,40,33	25,40,33
Nref	36644	36442
Tmin,Tmax	0.667,0.681	0.564,0.700
Tmin’	0.605	

```
Correction method= # Reported T Limits: Tmin=0.564 Tmax=0.700
AbsCorr = MULTI-SCAN

Data completeness= 0.994          Theta(max)= 66.596

R(reflections)= 0.1355( 21447)    wR2(reflections)= 0.4022( 36442)

S = 1.334                   Npar= 2449
```

The following ALERTS were generated. Each ALERT has the format
test-name_ALERT_alert-type_alert-level.
Click on the hyperlinks for more details of the test.

Alert level B

```
RFACR01_ALERT_3_B  The value of the weighted R factor is > 0.35
            Weighted R factor given   0.402
PLAT084_ALERT_3_B High wR2 Value (i.e. > 0.25) ...................       0.40 Report
PLAT220_ALERT_2_B Non-Solvent Resd 1   C   Ueq(max)/Ueq(min) Range        7.0 Ratio
PLAT306_ALERT_2_B Isolated Oxygen Atom (H-atoms Missing ?) .......        O1W Check
PLAT306_ALERT_2_B Isolated Oxygen Atom (H-atoms Missing ?) .......        O2W Check
PLAT306_ALERT_2_B Isolated Oxygen Atom (H-atoms Missing ?) .......        O3W Check
PLAT306_ALERT_2_B Isolated Oxygen Atom (H-atoms Missing ?) .......        O4W Check
PLAT306_ALERT_2_B Isolated Oxygen Atom (H-atoms Missing ?) .......        O5W Check
PLAT306_ALERT_2_B Isolated Oxygen Atom (H-atoms Missing ?) .......        O6W Check
PLAT306_ALERT_2_B Isolated Oxygen Atom (H-atoms Missing ?) .......        O7W Check
PLAT306_ALERT_2_B Isolated Oxygen Atom (H-atoms Missing ?) .......        O8W Check
PLAT306_ALERT_2_B Isolated Oxygen Atom (H-atoms Missing ?) .......        O9W Check
PLAT306_ALERT_2_B Isolated Oxygen Atom (H-atoms Missing ?) .......       O11W Check
PLAT430_ALERT_2_B Short Inter D...A Contact  O2W    ..  O9W     ..       2.74 Ang.
PLAT430_ALERT_2_B Short Inter D...A Contact  O3W    ..  O6C     ..       2.80 Ang.
PLAT430_ALERT_2_B Short Inter D...A Contact  O5W    ..  O11W    ..       2.61 Ang.
```

Alert level C

```
RFACG01_ALERT_3_C  The value of the R factor is > 0.10
            R factor given   0.135
RINTA01_ALERT_3_C  The value of Rint is greater than 0.12
            Rint given   0.153
PLAT020_ALERT_3_C The value of Rint is greater than 0.12 .........      0.153 Report
PLAT082_ALERT_2_C High R1 Value ..................................       0.14 Report
PLAT094_ALERT_2_C Ratio of Maximum / Minimum Residual Density ....       2.70 Report
PLAT220_ALERT_2_C Non-Solvent Resd 1   O   Ueq(max)/Ueq(min) Range        5.0 Ratio
PLAT222_ALERT_3_C Non-Solvent Resd 1   H Uiso(max)/Uiso(min) Range        7.8 Ratio
PLAT230_ALERT_2_C Hirshfeld Test Diff for    O6A    --  C53A    ..        6.6 s.u.
PLAT230_ALERT_2_C Hirshfeld Test Diff for    C45D   --  C46D    ..        5.9 s.u.
PLAT234_ALERT_4_C Large Hirshfeld Difference O2C    --  C46C    ..       0.19 Ang.
PLAT234_ALERT_4_C Large Hirshfeld Difference O2D    --  C46D    ..       0.19 Ang.
PLAT234_ALERT_4_C Large Hirshfeld Difference O4B    --  C49B    ..       0.20 Ang.
PLAT234_ALERT_4_C Large Hirshfeld Difference O4C    --  C49C    ..       0.18 Ang.
PLAT234_ALERT_4_C Large Hirshfeld Difference O6B    --  C52B    ..       0.20 Ang.
PLAT234_ALERT_4_C Large Hirshfeld Difference C45C   --  C46C    ..       0.19 Ang.
PLAT234_ALERT_4_C Large Hirshfeld Difference C48B   --  C49B    ..       0.20 Ang.
PLAT234_ALERT_4_C Large Hirshfeld Difference C48C   --  C49C    ..       0.16 Ang.
PLAT234_ALERT_4_C Large Hirshfeld Difference C51B   --  C52B    ..       0.16 Ang.
PLAT241_ALERT_2_C High   'MainMol' Ueq as Compared to Neighbors of        O6A Check
PLAT243_ALERT_4_C High   'Solvent' Ueq as Compared to Neighbors of        S4B Check
PLAT243_ALERT_4_C High   'Solvent' Ueq as Compared to Neighbors of        S7B Check
PLAT342_ALERT_3_C Low Bond Precision on  C-C Bonds ...............    0.01517 Ang.
PLAT413_ALERT_2_C Short Inter XH3 .. XHn     H47G   ..  H51B    ..       2.13 Ang.
```

Alert level G

```
PLAT002_ALERT_2_G Number of Distance or Angle Restraints on AtSite         76 Note
PLAT003_ALERT_2_G Number of Uiso or Uij Restrained non-H Atoms ...        272 Report
PLAT072_ALERT_2_G SHELXL First  Parameter in WGHT  Unusually Large       0.20 Report
PLAT175_ALERT_4_G The CIF-Embedded .res File Contains SAME Records          6 Report
PLAT176_ALERT_4_G The CIF-Embedded .res File Contains SADI Records          4 Report
PLAT178_ALERT_4_G The CIF-Embedded .res File Contains SIMU Records          4 Report
PLAT186_ALERT_4_G The CIF-Embedded .res File Contains ISOR Records          4 Report
```

```
PLAT199_ALERT_1_G Reported _cell_measurement_temperature ..... (K)       273 Check
PLAT200_ALERT_1_G Reported   _diffrn_ambient_temperature ..... (K)       273 Check
PLAT343_ALERT_2_G Unusual sp?      Angle Range in Main Residue for     C53B   Check
PLAT432_ALERT_2_G Short Inter X...Y Contact  O6D    ..  C3B     ..       2.93 Ang.
PLAT606_ALERT_4_G VERY LARGE Solvent Accessible VOID(S) in Structure        ! Info
PLAT720_ALERT_4_G Number of Unusual/Non-Standard Labels ..........          2 Note
PLAT790_ALERT_4_G Centre of Gravity not Within Unit Cell: Resd.  #          5 Note
              O
PLAT790_ALERT_4_G Centre of Gravity not Within Unit Cell: Resd.  #          7 Note
              O
PLAT790_ALERT_4_G Centre of Gravity not Within Unit Cell: Resd.  #          8 Note
              O
PLAT790_ALERT_4_G Centre of Gravity not Within Unit Cell: Resd.  #         10 Note
              O
PLAT790_ALERT_4_G Centre of Gravity not Within Unit Cell: Resd.  #         13 Note
              O
PLAT860_ALERT_3_G Number of Least-Squares Restraints .............       3712 Note
PLAT869_ALERT_4_G ALERTS Related to the use of SQUEEZE Suppressed           ! Info
```

---

0 **ALERT level A** = Most likely a serious problem - resolve or explain
16 **ALERT level B** = A potentially serious problem, consider carefully
23 **ALERT level C** = Check. Ensure it is not caused by an omission or oversight
20 **ALERT level G** = General information/check it is not something unexpected

2 ALERT type 1 CIF construction/syntax error, inconsistent or missing data
26 ALERT type 2 Indicator that the structure model may be wrong or deficient
8 ALERT type 3 Indicator that the structure quality may be low
23 ALERT type 4 Improvement, methodology, query or suggestion
0 ALERT type 5 Informative message, check

It is advisable to attempt to resolve as many as possible of the alerts in all categories. Often the minor alerts point to easily fixed oversights, errors and omissions in your CIF or refinement strategy, so attention to these fine details can be worthwhile. In order to resolve some of the more serious problems it may be necessary to carry out additional measurements or structure refinements. However, the purpose of your study may justify the reported deviations and the more serious of these should normally be commented upon in the discussion or experimental section of a paper or in the "special_details" fields of the CIF. checkCIF was carefully designed to identify outliers and unusual parameters, but every test has its limitations and alerts that are not important in a particular case may appear. Conversely, the absence of alerts does not guarantee there are no aspects of the results needing attention. It is up to the individual to critically assess their own results and, if necessary, seek expert advice.

Publication of your CIF in IUCr journals

A basic structural check has been run on your CIF. These basic checks will be run on all CIFs submitted for publication in IUCr journals (*Acta Crystallographica*, *Journal of Applied Crystallography*, *Journal of Synchrotron Radiation*); however, if you intend to submit to *Acta Crystallographica Section C* or *E* or *IUCrData*, you should make sure that full publication checks are run on the final version of your CIF prior to submission.

Publication of your CIF in other journals

Please refer to the *Notes for Authors* of the relevant journal for any special instructions relating to CIF submission.

PLATON version of 11/08/2016; check.def file version of 04/08/2016

Datablock 3_ftu1_s_sq - ellipsoid plot

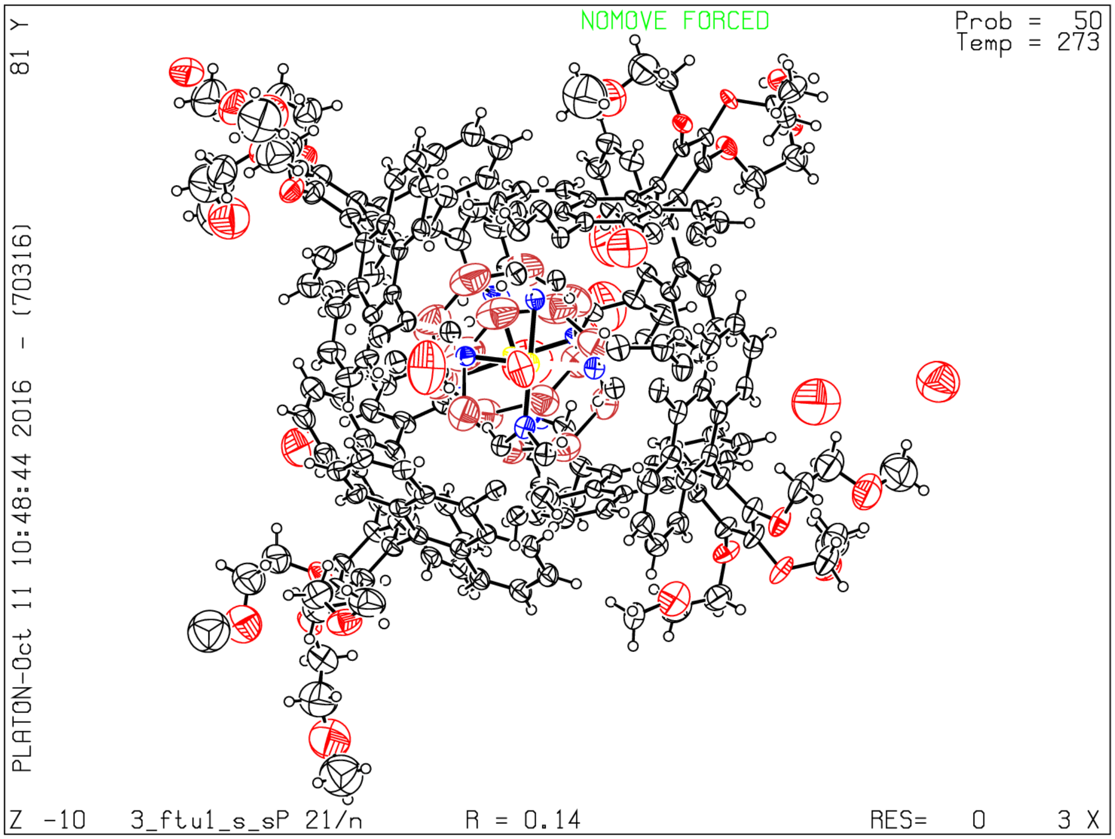

Supplement: Supplementary file 5 — Supplementary Data 3 [file 41467_2017_605_MOESM5_ESM.pdf]
