# Supplementary material for: Exact mass analysis of sulfur clusters upon encapsulation by a polyaromatic capsular matrix
Source: Nat Commun. 2017 Sep 29;8:749. doi: 10.1038/s41467-017-00605-5 (PMC5622087; doi:10.1038/s41467-017-00605-5)

checkCIF/PLATON report

You have not supplied any structure factors. As a result the full set of tests cannot be run.

THIS REPORT IS FOR GUIDANCE ONLY. IF USED AS PART OF A REVIEW PROCEDURE FOR PUBLICATION, IT SHOULD NOT REPLACE THE EXPERTISE OF AN EXPERIENCED CRYSTALLOGRAPHIC REFEREE.

No syntax errors found.   CIF dictionary   Interpreting this report

Datablock: SM321_H3

```
Bond precision:  C-C = 0.0200 A          Wavelength=0.71073

Cell:          a=21.297(2)       b=21.480(2)      c=27.276(3)
               alpha=70.017(1)   beta=69.062(1)   gamma=61.468(1)
Temperature:   90 K
```

	Calculated	Reported
Volume	10013.5(17)	10013.2(17)
Space group	P -1	P -1
Hall group	-P 1	-P 1
Moiety formula	C212 H184 N8 O24 Pd2, 2(S6), 4(N O3), 10(O)	?
Sum formula	C212 H184 N12 O46 Pd2 S12	C212 H184 N12 O46 Pd2 S12
Mr	4233.24	4233.22
Dx,g cm-3	1.404	1.404
Z	2	2
Mu (mm-1)	0.387	0.387
F000	4384.0	4384.0
F000’	4384.98	
h,k,lmax	22,23,29	22,23,29
Nref	26082	25841
Tmin,Tmax	0.973,0.985	0.761,0.985
Tmin’	0.962	

```
Correction method= # Reported T Limits: Tmin=0.761 Tmax=0.985
AbsCorr = MULTI-SCAN

Data completeness= 0.991          Theta(max)= 22.464

R(reflections)= 0.1346( 14302)    wR2(reflections)= 0.4282( 25841)

S = 1.060                  Npar= 2765
```

The following ALERTS were generated. Each ALERT has the format
test-name_ALERT_alert-type_alert-level.
Click on the hyperlinks for more details of the test.

Alert level A

```
THETM01_ALERT_3_A  The value of sine(theta_max)/wavelength is less than 0.550
            Calculated sin(theta_max)/wavelength =    0.5376
```

Alert level B

```
PLAT084_ALERT_3_B High wR2 Value (i.e. > 0.25) ...................       0.43 Report
PLAT220_ALERT_2_B Non-Solvent Resd 1   C   Ueq(max)/Ueq(min) Range        7.7 Ratio
PLAT306_ALERT_2_B Isolated Oxygen Atom (H-atoms Missing ?) .......        O1W Check
PLAT306_ALERT_2_B Isolated Oxygen Atom (H-atoms Missing ?) .......        O2W Check
PLAT306_ALERT_2_B Isolated Oxygen Atom (H-atoms Missing ?) .......        O3W Check
PLAT306_ALERT_2_B Isolated Oxygen Atom (H-atoms Missing ?) .......        O4W Check
PLAT306_ALERT_2_B Isolated Oxygen Atom (H-atoms Missing ?) .......        O5W Check
PLAT306_ALERT_2_B Isolated Oxygen Atom (H-atoms Missing ?) .......        O8W Check
PLAT306_ALERT_2_B Isolated Oxygen Atom (H-atoms Missing ?) .......        O9W Check
PLAT306_ALERT_2_B Isolated Oxygen Atom (H-atoms Missing ?) .......       O10W Check
PLAT306_ALERT_2_B Isolated Oxygen Atom (H-atoms Missing ?) .......       O11W Check
PLAT306_ALERT_2_B Isolated Oxygen Atom (H-atoms Missing ?) .......       O12W Check
PLAT430_ALERT_2_B Short Inter D...A Contact  O1W    ..  O1Z     ..       2.82 Ang.
PLAT430_ALERT_2_B Short Inter D...A Contact  O1Z    ..  O4W     ..       2.82 Ang.
PLAT430_ALERT_2_B Short Inter D...A Contact  O2W    ..  O5W     ..       2.77 Ang.
PLAT430_ALERT_2_B Short Inter D...A Contact  O3D    ..  O9W     ..       2.78 Ang.
PLAT430_ALERT_2_B Short Inter D...A Contact  O5W    ..  O8Z     ..       2.74 Ang.
PLAT430_ALERT_2_B Short Inter D...A Contact  O6A    ..  O12W    ..       2.77 Ang.
PLAT430_ALERT_2_B Short Inter D...A Contact  O6B    ..  O8W     ..       2.81 Ang.
PLAT430_ALERT_2_B Short Inter D...A Contact  O8W    ..  O6F     ..       2.60 Ang.
PLAT430_ALERT_2_B Short Inter D...A Contact  O9W    ..  O10W    ..       2.59 Ang.
PLAT430_ALERT_2_B Short Inter D...A Contact  O9Z    ..  O2H     ..       2.75 Ang.
```

Alert level C

```
REFNR01_ALERT_3_C  Ratio of reflections to parameters is < 10 for a
            centrosymmetric structure
            sine(theta)/lambda                 0.5376
            Proportion of unique data used     1.0000
            Ratio reflections to parameters    9.3458
PLAT082_ALERT_2_C High R1 Value ..................................       0.13 Report
PLAT088_ALERT_3_C Poor Data / Parameter Ratio ....................       9.43 Note
PLAT094_ALERT_2_C Ratio of Maximum / Minimum Residual Density ....       2.02 Report
PLAT220_ALERT_2_C Non-Solvent Resd 1   O   Ueq(max)/Ueq(min) Range        4.9 Ratio
PLAT222_ALERT_3_C Non-Solvent Resd 1   H Uiso(max)/Uiso(min) Range        8.6 Ratio
PLAT243_ALERT_4_C High   'Solvent' Ueq as Compared to Neighbors of        S7G Check
PLAT243_ALERT_4_C High   'Solvent' Ueq as Compared to Neighbors of       S11G Check
PLAT243_ALERT_4_C High   'Solvent' Ueq as Compared to Neighbors of        N4Z Check
PLAT244_ALERT_4_C Low    'Solvent' Ueq as Compared to Neighbors of        S8G Check
PLAT244_ALERT_4_C Low    'Solvent' Ueq as Compared to Neighbors of       S10G Check
PLAT244_ALERT_4_C Low    'Solvent' Ueq as Compared to Neighbors of        N2Z Check
PLAT342_ALERT_3_C Low Bond Precision on  C-C Bonds ...............       0.02 Ang.
PLAT369_ALERT_2_C Long    C(sp2)-C(sp2) Bond  C23B   -   C37B   ..       1.53 Ang.
PLAT369_ALERT_2_C Long    C(sp2)-C(sp2) Bond  C23C   -   C37C   ..       1.53 Ang.
PLAT412_ALERT_2_C Short Intra XH3 .. XHn     H49L   ..  H50L    ..       1.80 Ang.
PLAT413_ALERT_2_C Short Inter XH3 .. XHn     H46A   ..  H47L    ..       2.06 Ang.
PLAT430_ALERT_2_C Short Inter D...A Contact  O2W    ..  O11Z    ..       2.88 Ang.
PLAT601_ALERT_2_C Structure Contains Solvent Accessible VOIDS of .         60 Ang3
```

Alert level G

```
PLAT002_ALERT_2_G Number of Distance or Angle Restraints on AtSite       106 Note
PLAT003_ALERT_2_G Number of Uiso or Uij Restrained non-H Atoms ...       305 Report
PLAT072_ALERT_2_G SHELXL First  Parameter in WGHT  Unusually Large      0.30 Report
PLAT154_ALERT_1_G The s.u.'s on the Cell Angles are Equal ..(Note)     0.001 Degree
PLAT172_ALERT_4_G The CIF-Embedded .res File Contains DFIX Records         1 Report
PLAT175_ALERT_4_G The CIF-Embedded .res File Contains SAME Records         4 Report
PLAT176_ALERT_4_G The CIF-Embedded .res File Contains SADI Records         9 Report
PLAT178_ALERT_4_G The CIF-Embedded .res File Contains SIMU Records         2 Report
PLAT186_ALERT_4_G The CIF-Embedded .res File Contains ISOR Records         2 Report
PLAT301_ALERT_3_G Main Residue  Disorder ........... Percentage =          6 Note
PLAT302_ALERT_4_G Anion/Solvent Disorder ........... Percentage =         16 Note
PLAT304_ALERT_4_G Non-Integer Number of Atoms (   4.06) in Resd. #         2 Check
PLAT304_ALERT_4_G Non-Integer Number of Atoms (   1.94) in Resd. #         4 Check
PLAT333_ALERT_2_G Check Large Av C6-Ring C-C Dist. C10A   -C19A         1.42 Ang.
PLAT335_ALERT_2_G Check Large C6 Ring    C-C Range C12D   -C17D         0.21 Ang.
PLAT343_ALERT_2_G Unusual sp3      Angle Range in Main Residue for     C48D   Check
PLAT432_ALERT_2_G Short Inter X...Y Contact  O9Z    ..  C47H    ..      2.49 Ang.
PLAT432_ALERT_2_G Short Inter X...Y Contact  O11W   ..  C46H    ..      2.93 Ang.
PLAT432_ALERT_2_G Short Inter X...Y Contact  O12W   ..  C52D    ..      2.75 Ang.
PLAT432_ALERT_2_G Short Inter X...Y Contact  O12W   ..  C53D    ..      3.00 Ang.
PLAT432_ALERT_2_G Short Inter X...Y Contact  C14C   ..  C47H    ..      3.13 Ang.
PLAT432_ALERT_2_G Short Inter X...Y Contact  C14C   ..  C47B    ..      3.18 Ang.
PLAT432_ALERT_2_G Short Inter X...Y Contact  C15C   ..  C47H    ..      2.60 Ang.
PLAT432_ALERT_2_G Short Inter X...Y Contact  C15C   ..  C47B    ..      3.17 Ang.
PLAT432_ALERT_2_G Short Inter X...Y Contact  C16C   ..  C47H    ..      2.81 Ang.
PLAT432_ALERT_2_G Short Inter X...Y Contact  C34A   ..  C51D    ..      3.13 Ang.
PLAT720_ALERT_4_G Number of Unusual/Non-Standard Labels ..........         2 Note
PLAT790_ALERT_4_G Centre of Gravity not Within Unit Cell: Resd.  #         8 Note
              N O3
PLAT790_ALERT_4_G Centre of Gravity not Within Unit Cell: Resd.  #        13 Note
              O
PLAT790_ALERT_4_G Centre of Gravity not Within Unit Cell: Resd.  #        14 Note
              O
PLAT790_ALERT_4_G Centre of Gravity not Within Unit Cell: Resd.  #        17 Note
              O
PLAT860_ALERT_3_G Number of Least-Squares Restraints .............      4572 Note
```

---

  1 **ALERT level A** = Most likely a serious problem - resolve or explain
 22 **ALERT level B** = A potentially serious problem, consider carefully
 19 **ALERT level C** = Check. Ensure it is not caused by an omission or oversight
 32 **ALERT level G** = General information/check it is not something unexpected

```
  1 ALERT type 1 CIF construction/syntax error, inconsistent or missing data
 46 ALERT type 2 Indicator that the structure model may be wrong or deficient
  8 ALERT type 3 Indicator that the structure quality may be low
 19 ALERT type 4 Improvement, methodology, query or suggestion
  0 ALERT type 5 Informative message, check
```

It is advisable to attempt to resolve as many as possible of the alerts in all categories. Often the minor alerts point to easily fixed oversights, errors and omissions in your CIF or refinement strategy, so attention to these fine details can be worthwhile. In order to resolve some of the more serious problems it may be necessary to carry out additional measurements or structure refinements. However, the purpose of your study may justify the reported deviations and the more serious of these should normally be commented upon in the discussion or experimental section of a paper or in the "special_details" fields of the CIF. checkCIF was carefully designed to identify outliers and unusual parameters, but every test has its limitations and alerts that are not important in a particular case may appear. Conversely, the absence of alerts does not guarantee there are no aspects of the results needing attention. It is up to the individual to critically assess their own results and, if necessary, seek expert advice.

Publication of your CIF in IUCr journals

A basic structural check has been run on your CIF. These basic checks will be run on all CIFs submitted for publication in IUCr journals (*Acta Crystallographica*, *Journal of Applied Crystallography*, *Journal of Synchrotron Radiation*); however, if you intend to submit to *Acta Crystallographica Section C* or *E* or *IUCrData*, you should make sure that full publication checks are run on the final version of your CIF prior to submission.

Publication of your CIF in other journals

Please refer to the *Notes for Authors* of the relevant journal for any special instructions relating to CIF submission.

PLATON version of 11/08/2016; check.def file version of 04/08/2016

Datablock SM321_H3 - ellipsoid plot

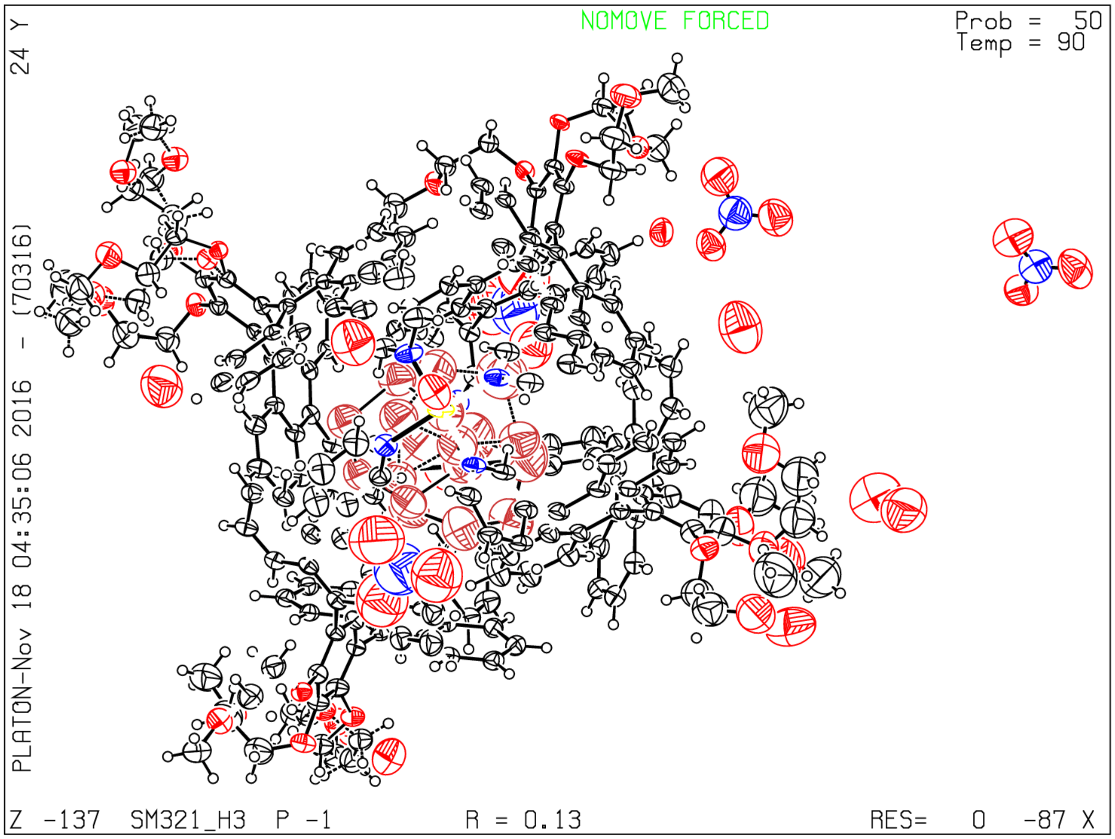

Supplement: Supplementary file 6 — Supplementary Data 4 [file 41467_2017_605_MOESM6_ESM.pdf]
